# Global disparities in surgeons’ workloads, academic engagement and rest periods: the on-calL shIft fOr geNEral SurgeonS (LIONESS) study

**DOI:** 10.1007/s13304-024-01859-7

**Published:** 2024-04-29

**Authors:** Mauro Podda, Marcello Di Martino, Francesco Pata, Giuseppe Nigri, Adolfo Pisanu, Salomone Di Saverio, Gianluca Pellino, Benedetto Ielpo, the LIONESS study group, Ashenafi Negash Tolla, Mohamed Abdelkhalek, Sameh Emile, Tesfaye Aga Dinagde, Chafik Bouzid, Ahmed Mohammed Farid Mahmoud Mansour, Ibrahim Umar Garzali, Alazar Berhe Aregawi, Tarek M. Zaghloul, Ali Kchaou, Selmy S. Awad, Amine Souadka, Tedla Gebretsadik Ted, Mohammad Saad Saumtally, Selmy S. Awad, Bensaad Ahmed, Nebiyou Simegnew Bayileyegn, Marco Nunziante, Mostafa Shalaby, Dawit Gebregiorgis Debena, Alemayehu Shanko Zena, Olayinka Lukman Adewunmi, Tolera A. Negasa, Mohamed Aboulkacem Bourguiba, Nura Feituri, Kirubel Abebe Kebede, Hossam Elfeki, Abdelhmed Anas, Ahmed K. Awad, Nebiyou Ermias, Dagnachew H. Hailemariam, Gosa Hundie Bejiga, Ruhama Yoseph Imana, Abebe Taffese, Tsega Terefe Feleke, Hosam Mohamed Elghadban, Bizuneh Sime Debela, Desta Shiferaw, Robel Tadesse, Isaac Sunday Chukwu, Abdullahi Ibrahim Mohammed, Khalid Arebo Hassen, Ndubuisi K. Obiagwu, Tadesse H. Tense, Mulugeta Taeme, Ewnte Biniam, Goytom Knfe Tesfay, Ayenachew Getachew Tegegn, Tewodros Abule Bilata Teddy, Bekana L. Bekana, Melkamu Kibret Nidaw, Mahmoud Ibrahim Ali, Kebebe Bekele Gonfa, Samuel Kiros, Tatek Belay, Gebeyaw Mengist Yallew, Chekol Wassie, Alem Mekete Ayalew, Fitsum Terefe Asfaw, Aimal Khan Aimal, Maria Agustina Casas, Fausto A. Madrid, Jose Antonio Diaz-Elizondo, Ignacio Guillermo Merlo, Tercio De Campos, Francisco Schlottmann, Carlos Augusto Gomes, Edgard Efrén Lozada Hernández, Augusto Moncada-Ortega, Antonio Ramos-De la Medina, Patricio Vanerio, Jose Antonio Diaz-Elizondo, Emilio J. Morales, Sarah Beth Stringfield, Cristian Gallardo, Vinicius Cordeiro Fonseca, Maria Emilia Muriel, Frederico Cantarino, Emilio J. Morales, Alessandro Fichera, Agustin E. Rodriguez, Roberto Paúl Andrade Salinas, Gonzalo Gabriel Crosbie, Carlos Augusto Metidieri Menegozzo, Nicolas Avellaneda, Cristhian Gonzalo Aspiazu Briones, Cristopher Varela, Warren E. Lichliter, Cristopher L. Varela, Darren Koppel, Jorge Godoy Lenz, Franco Pascual, Dauber A. Sibaja-Matamoros, Benjamin E. Johnson, Octavio Avila Mercado, Agustín Tita, Indraneil Mukherjee, Irving Lorenzo, Erick Rueda, Douglas Rico, Natasha Loria Terwes, Victor Visag-Castillo, Karol S. Escalante, Alan Francisco Alfaro Salvador, Luca Pio, Edgar B. Rodas, Juan Carlos Cardozo Aguilar, Ignacio Neri, Ernesto J. Barzola, Leonardo A. Barba, Andrés Mendoza Zuchini, Ana Karen Radilla Acevedo, Mahir Gachabayov, Ricardo Alessandro Teixeira Gonsaga, Fernando Bonilla, Chase Barrett Knickerbocker, Ioannis Tsouknidas, Juan C. Salamea, Lawrence Albert Salter Travers, Gustavo Adrian Nari, Diana Alejandra Pantoja Pachajoa, Michel Hernandez Valadez, Lyman Lansing Hale, Guillermo Perez, Asad Choudhry, Michaael L. Foreman, Ismael Brito, Mauricio Pedraza Ciro, Carla Joyce Newton, Hamilton Petry Souza, Daniel G. Davis, Rodrigo Vaz Ferreira, Deborah S. Keller, Gustavo Machain Miguel, Gurpreet Singh-Ranger, Neiling Massiel Oviedo Linares, Eduardo Smith Singares, Xavier HGarcía Valdivieso, Alfonso Ivan Sanchez Teran, Luis Felipe Cabrera Vargas, Agustín Valinoti, David M. Milne, Andrew Martin Fleming, Alejandro Quiroga-Garza, Eduardo Morales Espino, Nicole Mary Bedros, Geoffrey A. Funk, Fernanda V. S. Neves, Tommy Supit, Arshad Ahmed Baba, Mushtaq A. Chalkoo, Gultekin Ozan Kucuk, Ray Inocencio Sarmiento, Shamil I. Galeev, Mohammed J. Korkoman, Wah Yang, Noushif Medappil, Rahmatullah Athar, Ravichandran Subramaniam, Chi Fung Chia, Ahmad Alhazmi, Subhadip Halder, Siti Mayuha Rusli, Arshad Rashid, Barış Gülcü, Prasanth Nagarajan, Khaled Rida, Al-Radjid Jamiri, Oğuzkağan Batıkan, Huma Ahmed Khan, Ashrarur Rahman Mitul, Sergey Efetov, Marcelo A. F. Ribeiro Junior, Baris Gulcu, Marryam Riaz Farooqui, Venu Bhargava Mulpuri, Dinesh Bagaria, Ahmad Ramzi Yusoff, Rauf Wani, Muhammad Umar Younis, Krunal H. Khobragade, Muhammad Ibrahim Marwat, Lovenish Bains, Yoshiro Kobe, Peng Soon Koh, Umasankar Tantravahi, Ikhwan Sani Mohamad Mohamad, Hamdoon Abu-Arish, Ali Cihat Yildirim, Aini Fahriza Ibrahim, Jih Huei Tan, Dumaria Ketty Siagian, Neela Bhattacharya, Jennifer H. F. Chiu, Ganendra Paramasvaran, Kelvin Voon, Berk Göktepe, Lee Yeong Sing Lee, Amit Gupta, Faaiz Rasheed, Mohammed Al-Shehari, Syed Muhammad Ali Syed, Pawan Singh Bhat, Emre Erdoğan, Rajesh Roat, Aeris Jane D. Nacion, Swapnil Mukesh Saraiya, Akinwale Ibrahim Afolabi, Feng Yih Chai, Wagih Ghannam, Kaushik Bhattacharya, Harish NL, Ali Aloun, Hamza Waqar Bhatti, Akshadhar J. Koneti, Mohamad Klib, Ahmad Uzair Qureshi, Nicholas L. Syn, Ajaz Wani, Sara Saeidi, Muhammad Zeb, Kalyan Vijay Kumar Bandaru, Salomone Di Saverio, Maria Paola Menna, Salvador Morales-Conde, Emanuela Gessa, Santiago Sánchez-Cabús, Eleftherios Spartalis, Carmine Antropoli, Michele Manigrasso, Giuseppe S. Sica, Giorgio Ercolani, Andrei Chitul, Giulia Bacchiocchi, José Azevedo, Aleksandar Karamarkovic, Luigi Bonavina, Guglielmo N. Piozzi, Isabel Pascual Migueláñez, David Merlini, Piotr Major, Luciano Grimaldi, Valentin Calu, Aleksandar Karamarkovic, Giuseppe Curro, Christian Cotsoglou, Edoardo Baldini, Spiros Delis, Bojan Kovacevic, Michele Masetti, Francesco Selvaggi, Stefano Scabini, Andrian Reiti, Alessia Zullo, Antonio Giuliani, Pier Francesco Alesina, Lisa H. Massey, Alistair A. P. Slesser, Peter M. Neary, Kapil Sahnan, Jessica J. Tan, Gianluca Colucci, Richard R. W. Brady, Andrei Mihailescu, Michel Adamina, Ioannis Gerogiannis, Gabrielle van Ramshorst, Giulia Paradiso, Gianmario Edoardo Poto, Daniele Massaro, Fozia Nazir, Benjamin M. Stubbs, Francesco Menegon Tasselli, Virginia Durán Muñoz-Cruzado, Domenico Sabia, Alberto Vannelli, Valentina Enrica Marsella, Elisa Paoluzzi Tomada, Mauro Giambusso, Justin Davies, Aleksandar Karamarkovic, Alessandro Uzzau, Andrea Balla, Stefano Scaringi, Fozia Nazir, Francesco Lancellotti, Eleftheria Douka, Giulio Argenio, Stefano Scaringi, Gianluca Cassese, Michele Ammendola, Luca Morelli, Elisa Francone, Miklosh Bala, Prem Thambi, Maurizio Iacobone, Andrea Campisi, Patricia Tejedor, Gabriele Spoletini, Matteo Mascherini, Francesco Belia, Kapil Sahnan, Alessandro Uzzau, Ufuk Oguz Idiz, Elisa Francone, Elif Colak, Paul Adewunmi Peters, Gianluca Pellino, Emilio Peña Ros, Erman Aytac, Ramon Vilallonga, Alejandro Serrablo, Edoardo Maria Muttillo, Gregorio Di Franco, Gianluca Vanni, Arda Isik, Valentina Sbacco, Francesca Paola Tropeano, Iride Porcellini, Rebecca Jayne Butler, Carmen Cagigas Fernandez, Aristeidis Papadopoulos, Silvia Salvans, Jan Sher Khan, Pedro Palazón Bellver, Sebastian Jeri Mc Farlane, Konstantinos Apostolou, Jaume Tur-Martinez, Javier Rivera Castellano, Matteo Santoliquido, Luis Tallon-Aguilar, Valerio Celentano, Vusal Aliyev, Andrei Mihailescu, Issam al-Najami, Florin A. Grama, Jaume Tur-Martinez, Łukasz Masior, Wojciech J. Ciesielski, Antonio Luberto, Marco Sparavigna, Tomasz Jerzy Klimczak, Ionut Negoi, Mario Serradilla-Martín, Athanasios Marinis, Roberto Cammarat, Juan Bellido-luque, Carlo Alberto Manzo, Flavio Milana, Gaetano Luglio, Narcis Octavian Zarnescu, Isidro Martínez, Stefano Olmi, Radu V. Costea, Carlos Cerdán-Santacruz, Nicolae Gica, Gianluca Rompianesi, Gaetano Gallo, Cristina Ridolfi, Saidah Sahid, Tommaso CC, Guillermo López-Soler, Carlos JGómez Díaz, Niamh Mccawley, Andrea Pierre Luzzi, David Abelló, Alba Correa Bonito, Vasileia Ntomi, Stavros Gourgiotis, Natalia Suárez Pazos, Marta Jiménez Toscano, David Ambrona Zafra, Marc Beisani, Alba Oliva, Mert Guler, Marta Jiménez Gómez, Lidia Oddis, Blanca Montcusí, Ander Timoteo Delgado, Mateusz PJęckowski Aingeru Sarriugarte Lasarte, Nicola Antonacci, Alberto Sartori, John Vincent Taylor, Francisco Juan de Santos Iglesias, Manuela Mastronardi, Emre Gunay, Andrew Yiu, Oscar Cano-Valderrama, Giulia Costantini, Holly Jane Digne-Malcolm, Thalia Petropoulou, Marta Jiménez Gómez, Nir Horesh, Michael E. Kelly, Nauman Ahmed, Justyna Wyroslak-Najs, Prashant Naik, Vincenzo Vigorita, Michael Michael, Ernest Castro, Anna Paspala, Marco D’Ambrosio, Anang Pangeni, Jana Dziakova, Flavio Milana, Lara Blanco Terés, Leyre Lorente-Poch, Isidoro Di Carlo, Michael Spartalis, David Moro-Valdezate, Matthew G. Davey, Victor Turrado-Rodriguez, Yegor Tryliskyy, Marco Calussi, Silvia Pérez Farré, Miriam Cazador Labat, Chiara Marafante, Marijn Takkenberg, Charlotte Elisabeth Brookes, Antonio S. Soares, Eleftherios Gialamas, Damiano Caputo, Laia Codina Corrons, Nicola Antonacci, Sante Capitano, Alexandros Charalabopoulos, Cristine Brooke Pathirannehalage Don, Enrico Marrano, Maria Carmela Giuffrida, Giovanni Tomasicchio, Vittoria Bellato, Jasper Stijns, Ben Creavin, Dimitrios Schizas, Francisco Blanco-Antona, Stylianos Kykalos, Nicola Tartaglia, Amalia Pelegrina, Alberto G. Barranquero, Carlo Alberto Schena, Simone Cremona, Efstratia Baili, Pasquale Cianci, Annamaria Di Bella, Edoardo Forcignanò, Tiziana Fabbrizio, Francesco Salvetti, Marco Inama, Tommaso Campagnaro, Eva B. Deerenberg, Isabella Reccia, Aldo Rocca, Federico Fazio, Athanasios Tampakis, Kostas Tepelenis, Giovanna Di Meo, Georgios Konstantoudakis, Dimitrios K. Manatakis, Alexandros Charalabopoulos, Michela Mineccia, Antonella Delvecchio, Carmelo Lo Faro, Edoardo Rosso, Samik K. Bandyopadhyay, Michael Queffurus, Jacopo Nicolò Marin, Arianna Corvasce, Gauri Chillarge, Mohammed Mustafa Mohammed, Rebecca Reid, Dario Gherardi, Awais Naeem, Sanjay Pandanaboyana, Salim Tayeh, Mickael Chevallay, Gregory Sergeant, Salvatore Cuccomarino, Nick Salimian, Panagiotis Kapsampelis, Carlotta Ferretti, James Glasbey, Rafique Umer Harvitkar, Muhammad Rafaih Iqbal, Gabriel Paiva de Oliveira, Sonia Pérez-Bertólez, Alessandro Bergna, Giuseppe Brisinda, Matteo Rottoli, Francesco Roscio, Corrado Pedrazzani, Jeremy Meyer, Marco V. Marino, Camille Brasset, Clerc Daniel, Stefanie Devriendt, Gabriele Soldini, Damiano Pennisi, Chris B. Richards, Felicia Fiore, Antonio Bocchino, Stephanie Au, Miguel Hernandez-Garcia, Alice Frontali, Ugo Grossi, Evangelos Lolis, Marco Miotto, Giacomo Di Filippo, Charalampos Seretis, Andrea Barberis, Raffaele De Luca, Giovanni Battista Levi Sandri, Elisa Reitano, Marco Mattioli, Sergey Efetov, Guido Sciaudone, Giacomo Fuschillo, Boris Schiltz, Juin Low, Georgios Bointas, Valerio Cozza, Vaihere Delaune, Marco D’Ambrosio, Francesca Vescio, Silvia Curcio, Husnu Sevik, Pietro Santocchi, Tommaso Stecca, Alexandre Balaphas, Antonella Chessa, Cosimo Alex Leo, Fabio F. di Mola, Mariarita Tarallo, Francesco Martini, Giorgio Lisi, Mansoor Khan, Marco Nicolazzi, Enrico Pinotti, Andrea Morini, Giuseppe Esposito, Oreste Claudio Buonomo, Diletta Corallino, Erman Aytac, Anıl Demir, Raffaello Roesel, Chiara De Lucia, Michele Maruccia, Oguzhan Tekin, Giovanna Pavone, Ashish Gupta, Irene Pradelle, Michael Chrysikos, Maira Farrukh, Gian Luca Baiocchi, Carla Piras, Antonio Melero Abellán, Laura Olivieri, Marco Materazzo, Ludovico Carbone, Audrius Dulskas, Francesk Mulita, Federico Corronca, Micaela Cappuccio, Francesco Menegon Tasselli, Elia Smerieri, Maria Sotiropoulu, Tommaso Violante, Lucia Moletta, Martin Rutegård, Antonio Brillantino, Francesco Pata, Aitor Landaluce-Olavarria, Paolo Ossola, Dario Bono, Helena Salvador Rosés, Luis E. Perez-Sanchez, Sonia Sabbatini, Mauro Alessandro Scotti, Roberto Cammarata, Noelia Ibáñez, Marco Materazzo, Alessandra Marano, Silvia Palmisano, Pedro Cascales-Sanchez, Vinicio Mosca, Cristina Ballester, Madalina Ionela Iordache-Petrescu, Gioia Pozza, Omer Akay, Ruggero Bollino, Gianluca Vanni, Johannes M. A. Toti, Gianluca Mascianà, Radoslaw Pach, Bruno Sensi, Savvala N. Natalia, Alessandro Coppola, Yuksel Altinel, Francesco Brucchi, Marc Pérez Guitart, Andrea Jiménez Salido, Nicolò Tamini, Zoe Garoufalia, Patricia Mifsut, Alex Wilkins, Antonio Navarro-Sánchez, Giovanni Cestaro, Luigi Conti, João M. B. Carvas, Carlo Gazia, Patricia Alonso, Giovanni Spiezio, Benedetto Ielpo, Giulia Maggi, Jovan Juloski, Bartolomeo Braccio, Clara Pañella, Celia Caula Freixa, Georgios Petrakis, Cristina Folliero, Beatriz De Andrés-Asenjo, Alessandra Iodice, Hannes Jansson, Tomas Elosua González, Irene Ortega, Maria J. Castro, Agostino Fernicola, Antonio Carlos Maya Aparicio, Nuria Ortega, Christos Chouliaras, Marina Vila Tura, Zacharoula Sidiropoulou, Ana Gonzalez, Goran Augustin, Juan J. Segura-Sampedro, Vladica Cuk, Gaetano Piccolo, Sara Ingallinella, Monica Ortenzi, Fabio Uggeri, Salvatore Carrabetta, Matti Tolonen, Giampaolo Formisano, Simone Manfredelli, Teresa Perra, Francesco Esposito, Gennaro Perrone, Georgios Zacharis, Jaime Iturbe Menéndez, Sofia Maria Jaume Bottcher, Orestis Ioannidis, Nuno Borges, Xavier Sousa, Renan C. Colombari, Clara Tellez, Gabriella Teresa Capolupo, Giuseppe Giuliani, Federica Mastella, Lluis García González, Ilenia Merlini, Federico Ghignone, Tiago Correia de Sa, Orsalia Mangana, Mario Rodriguez-Lopez, Mario Alvarez-Gallego, Ashim Chowdhury, Pierpaolo Di Lascio, Virginia Jiménez Carneros, Marco Angrisani, Luigi E. Conte, Carmen Galiana Montiel, Francesca Ascari, Irida Dajti, Pierfrancesco Lapolla, Beatrice Sperotto, Gennaro Mazzarella, Marianna Capuano, Maria Martinez Lopez, Luca Ferrario, Joris P. Bulte, María Martínez López, Claudio Guerci, Angelo Alessandro Marra, Acidi Belkacem, Raquel Escalera Pérez, Arpád Panyko, Evgeni Dimitrov, Eduardo J. Luque, Mario Montes Manrique, Mykola Paranyak, Mario Montes Manrique, Mandeep Kaur, Giuliano Barugola, Christos Farazi-Chongouki, Mercedes Estaire-Gómez, Juan Carlos Martín del Olmo, Bruno Nardo, Arda Ulaş Mutlu, Ainhoa Valle Rubio, David A. Merlini, Francesco Marchegiani, Marco Angelo Cereda, Danilo Vinci, Iman Komaei, Niccolo Petrucciani, Steffen Seyfried, Petr Krsicka, Fabio Medas, Dusan Lesko, Valentina Murzi, Simone Manfredelli, Leandro Siragusa, María Alejandra Guerrero, Marina Alarcón Iranzo, Luca Cestino, Alessandro Pasculli, Nazareno Smerieri, Ilaria Benzoni, Federico Festa, Antonio Brillantino, Daniel Filipe Martins Jordão, Eugenio Licardie, Diana C. Nicolaescu, Valerio Balassone, Vincenzo La Vaccara, Fabrizio Romano, Dmitry Mikhailovich Adamovich, Panayiotis Papatheodorou, Giuseppe Nigri, Norvana Maroni, Enrique Colás-Ruiz, Liene Melberga, Federica Chimenti, Davide Ferrari, Claudio Arcudi, Andrea Police, Belinda De Simone, Luis Sánchez-Guillén, Felipe Carlos Parreño-Manchado, Juan José Rachadell, Diego Coletta, Azize Saroglu, Gianmaria Casoni Pattacini, Amedeo Antonelli, Marco Anania, Francesco Litta, Stefano Lafranceschina, Andrea Bottari, Mladen Pavlovic, Noel Rojas-Bonet, Ekaterini Christina Tampaki, Jurij A. Kosir, Ferdinando M. Anelli, Sara Gortázar de las Casas, Rabia Kucukarslan, Elisa Sefora Pierobon, Maria Milanesi, Maria Drogouti, Maria Sotiropoulou, Federica Di Marco, Elisa Bannone, Svenja Christin Sliwinski, Ishak Yildiz, Marco Pellicciaro, Andrea Celotti, Michele Malerba, Fabio Marino, Eirini Bourmpouteli, Giuseppe Bianco, Evgenia Charitaki, Marco Maria Pascale, Argyrios Ioannidis, Fatlum Maraska, Lorenzo Petagna, Giulia Turri, Michail Vailas, Andrea Romanzi, Sjaak Pouwels, Fausto Rosa, Lucrezia Clocchiatti, Fausto Rosa, Jacopo Andreuccetti, Nikolaos Machairas, Maximos Frountzas, Konstantinos Lasithiotakis, Mirko Barone, Prokopis Christodoulou, Dario Potkonjak, Alberto Aiolfi, Hilbert S. de Vries, Sara Napetti, Antonio Castaldi, Pedro Botelho, Irene Marziali, Matteo Uccelli, Michele Pisano, Massimiliano Veroux, Eleonora Locci, Angela Romano, Mariagiulia Dal Cero, Pierfranco Maria Cicerchia, Caterina Baldi, Iris Shari Russo, Silvia Maria Tenconi, Michele Malerba, Fatmir Saliu, Andrea Bondurri, Luca Cardinali, Giovanni Guglielmo Laracca, Beatriz Martín-Pérez, Mauro Alessandro Scotti, Daniel M. Felsenreich, Miguel Cunha, Domenico Zerbo, Marco Clementi, Daniele Delogu, Angelo Iossa, Akshay Prasannakumar Bavikatte, Can Saracoglu, Federica Di Marco, Nicolò Pecorelli, Andrea Tufo, Carmelo Lo Faro, Alan Biloslavo, Georgios D. Lianos, Filippo Carannante, Antonio Costanzo, Mauro Montuori, Maria Cigognini, Roberto Silvestro, Semra Demirli Atici, Pamela Milito, Antonio Ferronetti, Mert Güngör, Antonella Chessa, Beatrice Di Venere, Caterina Puccioni, Marco Calussi, Giovanni De Nobili, Lucio Selvaggi, Tommaso Farolfi, Pauline Aeschbacher, James B. Olivier, Giuseppe Frazzetta, Valeria Andriola, Alessio Giordano, Alessandro De Luca, Francesco Ferrara, Biagio Casagranda, Fabrizio Sammartano, Alfio Alessandro Russo, Stefano Rossi, Mario Pacilli, Eirini Synekidou, Roberta Tutino, Marco Ceresoli, Michele De Rosa, Andrea G. Di Santo Albini, Paolo Vincenzi, Samantha Vellei, Enrico Benzoni, Gabriela Aracelly Arroyo Murillo, Giuliano Izzo, Bruno Scotto, Riccardo Caruso, Francesco Colombo, Fabrizio D’Acapito, Eftychios Lostoridis, Edoardo Saladino, Gaetano Poillucci, Beat Moeckli, Massimiliano Veroux, Ottavia Manto, Yasin Kara, Cihad Tatar, Stefano Piero Bernardo Cioffi, Sara Mambrilla, Valentina Bianchi, Sara Mambrilla, Michele Scopelliti, Ferdinando Agresta, Valeria Andriola, Andrea Kazemi Nava, Marcello Di Martino, Eloisa Franchi, Pietro Fransvea, Mikel Prieto, Biagio Picardi, Antonia Rizzuto, Paolo Panaccio, Stefano Gussago, Ester Marra, Pietro Addeo, June Fernández Fernández, Mehmet Gulmez, Donatella Pisaniello, Maria Paola Menna, Maurizio Cannavo, Victor López-López, Pasquale Avella, Andrea Peloso, Lidia Oddis, Giovanni Bellanova, Giuseppe Candilio, Bruno Perotti, Roberta Granata, Gaetano Florio, Raffaello Roesel, Giovanna Ioia, Immacolata Iannone, Giorgio Lisi, Giorgio Ammerata, Martina Zambon, Francesco Maria Romano, Simone Rossi Del Monte, Diego Sasia, Laura Fortuna, Mario Giuffrida, Andrey Litvin, Marco Cannistra, Cristina De Padua, Tatiana Gómez Sánchez, Sara Ingallinella, Marta Paniagua-Garcia-Señorans, Jenny Guevara, Arkaitz Perfecto, Felipe Alconchel, Michela Campanelli, Laura Keci, Michele Cricrì, Georgios Kampouroglou, Giulia Bagaglini, Sara Sentí Farrarons, Antonio Greco, Maciej Walędziak, João M. Carvas, Monica Serrano-Navidad, Berend Van Doorn, Ciro Schiavo, Nicolò Fabbri, Ishfaq Ahmad Wani, Isabella Mondi, Antonio Castaldi, Elissavet Anestiadou, Beatriz ISilva Mendes, Daunia Verdi, Panagiotis Dorovinis, Danilo Vinci, Andrea Benedetti Cacciaguerra, Federica Di Marco, Beatriz Caldeira, Giuseppe Canonico, Dragomir Dardanov, Stefano D’Ugo, Fayez Maged Francis, Simona Meneghini, Antonio Pesce, Silvana Bernadetta Puglisi, Maria Irene Bellini, Marcello Pisano, Marco Giordano, Marianna Capuano, Francesco Fleres, Federica Campus, Nicola Cillara, Elisabetta Moggia, Francesco A. Ciarleglio, Mauro Podda, Giacomo Calini, Gianpiero Gravante, Riccardo De Carlis, Michele Altomare, Gennaro Martines, Marco Miggino, Luigi Marano, Alberto Stocco, Alberto Porcu, Thuy Vy Pham, Maria Clelia Gervasi, Tiziana Pilia, Giuseppe Palomba, Andrea Picchetto, Marcello Giuseppe Spampinato, Marco Cannistra, Michail Vailas, Francesco Tandoi, Micaela Piccoli, Malina ECaraiman Gall, Andrew Yiu, Audrius Dulskas, Bárbara Ribeiro Santos, Ervis Agastra, Tijmen Koëter, Elgun Samadov, Patrizia Marsanic, De Fatico Gilda Serena, Mustafa Yener Uzunoglu, Giacomo Zanus, Eriol Braholli, Gennaro Giovine, Giampaolo Galiffa, Sara Lauricella, Saidrakhim Lukmonov, Sezai Leventoglu, Serge Chooklin, Nagendra N. Dudi-Venkata, Chahaya M. Gauci

**Affiliations:** 1 Department of Surgical Science, Emergency Surgery Unit, Policlinico Universitario “D. Casula”, Azienda Ospedaliero-Universitaria di Cagliari, University of Cagliari, SS 554, Km 4,500, 09042 Cagliari, Monserrato Italy; 2 Department of Health Sciences, Università del Piemonte Orientale, Novara, Italy; 3 Department of Pharmacy, Health and Nutritional Sciences, University of Calabria, Rende, Italy; 4 Department of Medical-Surgical Sciences and Translational Medicine, Sapienza University of Rome, Rome, Italy; 5 Department of Surgery, Madonna del Soccorso Hospital, San Benedetto del Tronto, Italy; 6 Department of Colorectal Surgery, Vall d’Hebron University Hospital, Universitat Autonoma de Barcelona UAB, Barcelona, Spain; 7 Department of Advanced Medical and Surgical Sciences, Universitá degli Studi della Campania ‘Luigi Vanvitelli’, Naples, Italy; 8 Hepatobiliary Surgery Unit, Hospital del Mar, University Pompeu Fabra, Barcelona, Spain

**Keywords:** Surgeon’s workload, On-call, Surgeon’s well-being, Human Development Index, Global research

## Abstract

The workload of general surgeons is multifaceted, encompassing not only surgical procedures but also a myriad of other responsibilities. From April to May 2023, we conducted a CHERRIES-compliant internet-based survey analyzing clinical practice, academic engagement, and post-on-call rest. The questionnaire featured six sections with 35 questions. Statistical analysis used Chi-square tests, ANOVA, and logistic regression (SPSS^®^ v. 28). The survey received a total of 1.046 responses (65.4%). Over 78.0% of responders came from Europe, 65.1% came from a general surgery unit; 92.8% of European and 87.5% of North American respondents were involved in research, compared to 71.7% in Africa. Europe led in publishing research studies (6.6 ± 8.6 yearly). Teaching involvement was high in North America (100%) and Africa (91.7%). Surgeons reported an average of 6.7 ± 4.9 on-call shifts per month, with European and North American surgeons experiencing 6.5 ± 4.9 and 7.8 ± 4.1 on-calls monthly, respectively. African surgeons had the highest on-call frequency (8.7 ± 6.1). Post-on-call, only 35.1% of respondents received a day off. Europeans were most likely (40%) to have a day off, while African surgeons were least likely (6.7%). On the adjusted multivariable analysis HDI (Human Development Index) (aOR 1.993) hospital capacity > 400 beds (aOR 2.423), working in a specialty surgery unit (aOR 2.087), and making the on-call in-house (aOR 5.446), significantly predicted the likelihood of having a day off after an on-call shift. Our study revealed critical insights into the disparities in workload, access to research, and professional opportunities for surgeons across different continents, underscored by the HDI.

**Supplementary Information:**

The online version contains supplementary material available at 10.1007/s13304-024-01859-7.

## Introduction

The workload of a general surgeon is multifaceted, encompassing not only surgical procedures but also a myriad of other responsibilities [1–5]. High workloads and insufficient recovery time can harm surgeons and patients, impacting the quality of care, outcomes, and surgeon’s well-being [6].

Surgical outcomes, quality of complex decision-making, and academic tasks are influenced by alertness, reaction time, eye–hand coordination, and concentration. While there is increasing focus on patient safety and adverse events because of medical errors [7, 8], less attention has been paid to surgeons’ well-being [9, 10]. This started a debate on how doctors should handle post-call fatigue and elective surgery after on-call. Several suggestions have been made [11], including that surgeons have ethical obligations to sleep and be well-rested to perform safe surgery [12].

*Surgeon’s workload and the opportunity to access research might be influenced by resources, patient demographics, professional development opportunities, societal expectations, and governmental support. However,* the role of such agents needs to be elucidated [13]*. In an era where the sustainability of healthcare systems is under scrutiny, there is a clear need to better understand these dynamics and develop effective strategies to manage surgeon workloads and professional development at a global level. The present large-scale, international study aimed at filling this gap, analyzing the interrelationships between clinical workload, academic engagement, on-call shifts, and post-on-call rest. It also assessed the potential association between workload and local factors.*

## Methods

The Association of Italian Surgeons in Europe (ACIE), in collaboration with Association of Italian Surgeons in North America (AISNA) and Italian Surgical Research Group (ItSURG), conducted an internet-based cross-sectional survey to detect differences, strengths, and limitations among different national health systems concerning general surgery activity.

A brief informative letter about the study was shared with 1.600 surgeons. Informed consent of individuals was obtained with Google Forms. Survey respondents were informed of the purpose of the study, and their participation remained voluntary as no incentives were offered.

This survey was developed and reported as per the Checklist for Reporting Results of Internet E-Surveys (CHERRIES) [14]. For this research, which involved a non-intrusive, anonymized web-based survey, no formal ethical approval was deemed necessary.

### Questionnaire development and composition

The Steering Committee developed the questionnaire using web-based and remote discussions after identifying the components and topics to include. The questionnaire underwent a preliminary beta testing phase before its official deployment to ensure its comprehensibility, relevance, and user-friendliness. Once agreement had been reached, the questionnaire (The On-calL shIft fOr GeNEral SurgeonS LIONESS Study Form) was completed using Google Form survey software (Google, Mountain View, California, USA).

The questionnaire had six sections and 35 questions (Supplementary Material Table 1). Both closed-ended (19/35) and open questions (16/35) were used. The first four sections included general questions about the baseline characteristics of the respondents, country and continent of practice, type of sub-specialty, years of practice, practice level, and hospital organization. The other sections focused on the type of clinical practice, research activity, and on-call shifts.

### Study circulation

On April 1st 2023, the questionnaire was made available online and was open for completion until May 15th 2023. The survey (https://docs.google.com/forms/d/e/1FAIpQLSerFw4p_T2ADKdwkacN-SiPLyevO0KOP1PbO_esLkJxVVZqXg/viewform?usp=sf_link) was disseminated through general surgeons and trainees within the ACIE, AISNA and ItSurg networks. Three reminder emails were sent at three-week intervals, encouraging participants who had not yet responded to complete the survey.

### Participants

Participants consisted of general surgeons comprising surgical trainees and fellows (classified as *trainee* for the planned analyses) and consultant/attending surgeons, researchers/professors, and directors (classified as *consultant/professor* for the planned analyses) currently covering on-call shifts in their hospitals across Europe, Asia, Africa, Oceania, North and South America. We excluded specialists in other surgical fields, such as gynecology, vascular surgery, head and neck surgery, cardiac and thoracic surgery, neurosurgery, and urology.

### Data handling and extraction

A Steering Committee member (MP) downloaded the questionnaires and shared them with the other members for data analysis and discussion. Multiple entries from the same individual were sought manually and eliminated.

### Domains assessed

- **Clinical practice**: number of on-call duties per month, duration of each on-call shift, number of patients managed during an on-call shift.
- **Academic engagement**: number of scientific articles published per year, number of scientific articles read per month, frequency of attending scientific conferences as a faculty member per year, number of scientific conferences attended per year in a learning capacity.
- **Post-on-call rest**: the provision of a day off following an on-call shift or two days off if on-call during public holidays.

### Statistical analysis

Categorical variables were reported using counts and percentages, while continuous variables were reported by means and standard deviation or median and interquartile (IQR), depending on the type of distribution. Variables were stratified between the two different levels of practice (*trainee *vs* consultant/professor*) and continents, compared using 4 × 2 or 5 × 5 contingency tables, and analyzed using the Chi-square test for categorical or one-way analyses of variance (ANOVA) for continuous variables, respectively. Univariable logistic regression analysis examined the association between variables and providing a day off following an on-call shift.

All variables associated with the provision of a day off following an on-call shift at a threshold of p-values less than 0.05 in the univariable logistic regression analyses were entered in the final multivariable model. Multivariable logistic regression analyses with backward stepwise elimination tested which variables had the best ability to explain the chance of having a day off after an on-call shift.

Results were considered statistically significant if *p*-values were < 0.05. SPSS^®^ version 28 (IBM, Armonk, New York, USA) was used for the statistical analysis.

Different subgroup analyses were planned to aim at exploring possible variations in practice and workload of surgeons based on the level of practice, length of the on-call shifts (> 12 h), different continents, and different Human Development Index (HDI) (retrieved from https://hdr.undp.org/data-center/human-development-index#/indicies/HDI). HDI is a composite measure including the health (life expectancy at birth), education (mean years of schooling for adults and expected years of schooling for children), and the standard of living (per capita income indicators) dimensions commonly used to measure a country’s development by the United Nations Development Programme [13]. The countries, classified into four tiers based on the HDI, were categorized as having a high HDI, very high HDI, or a non-very-high HDI to ensure a balanced comparison among the groups.

## Results

### Baseline characteristics of the respondents

**Fig. 1:**
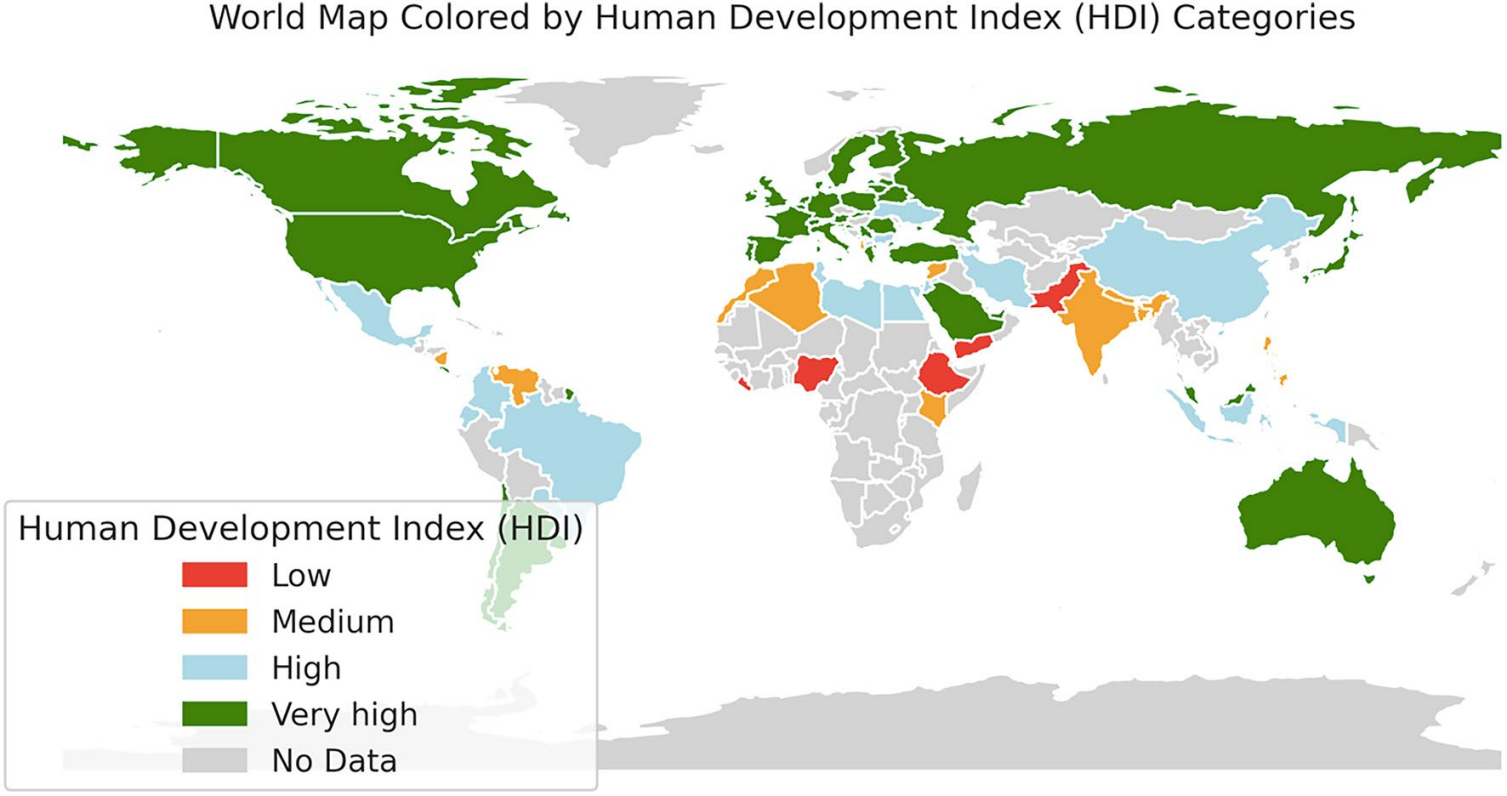
Global distribution of survey respondents by Human Development Index (HDI) categories. This map illustrates the geographic dispersion of participants in our survey, categorized by the HDI of their respective countries. Areas are color-coded to represent different levels of development: very high (dark green), high (light green), medium (orange), low (red), and regions with no data (grey)

Our survey was distributed to approximately 1.600 surgeons. We received a total of 1.046 responses (65.4%) (Table 1), of whom 40.2% were based in Italy and 11.8% in Spain. Most (78.1%) respondents were from Europe and 65.1% worked at a general surgery unit. Participating surgeons were general (34.4%), colorectal (32.2%), HPB (12.4%), and emergency surgeons (7.9%). Overall, they had a duration of surgical activity lasting 10.7 years. Responders mainly came from public teaching hospitals (75.9%) with more than 400 beds (47.6%). The global distribution of respondents by HDI categories is shown in Fig. 1.

**Table 1:** Baseline characteristics and study results

	Overall	Europe	North America	South America	Africa	Asia	Oceania	*p* value
Responders	1,046	817 (78.1%)	24 (2.3%)	69 (6.6%)	60 (5.7%)	76 (7.2%)	2 (0.2%)	
Male sex	781 (74.7%)	576 (70.5%)	20 (83.3%)	61 (88.4%)	58 (96.7%)	66 (86.8%)	2 (100%)	< 0.001
Mean age	39.0 ± 8.3 37.0 (IQR 10)	38.8 ± 8.2 37.0 (IQR 10)	43.9 ± 11.6 40.5 (IQR 16)	41.2 ± 9.2 39.0 (IQR 11)	36.1 ± 4.7 35.0 (IQR 5)	39.9 ± 7.9 39.5 (IQR 8)	35.0 ± 4.2 35.0 (IQR 3)	0.001
N. Working in Subspecialty units (only one subspecialty present)	362 (34.9%)	287 (35.1%)	13 (54.2%)	26 (37.7%)	17 (28.3%)	21 (27.6%)	2 (100%)	0.131
N. Working in General surgery units (more than one subspecialty present)	681 (65.1%)	530 (64.9%)	11 (45.8%)	43 (62.3%)	43 (71.7%)	55 (72.4%)	–	0.131
Type of specialty								
General surgery	360 (34.3%)	242 (29.6%)	9 (37.5%)	33 (47.8%)	44 (73.3%)	32 (42.1%)	1 (50%)	< 0.001
Colorectal	336 (32.2%)	292 (35.7%)	9 (37.5%)	11 (15.9%)	6 (10.0%)	19 (25.1%)	1 (50%)	< 0.001
Endocrine	16 (1.5%)	14 (1.7%)	–	–	1 (1.7%)	1 (1.3%)	–	0.927
Hepatopancreatobiliary (HPB)	129 (12.4%)	110 (13.5%)	–	6 (8.7%)	4 (6.7%)	10 (13.2%)	–	0.285
Upper GI	62 (6.0%)	49 (6.0%)	–	10 (14.6%)	1 (1.7%)	3 (3.9%)	–	0.023
Breast	21 (2.0%)	17 (2.1%)	–	–	2 (3.3%)	2 (2.6%)	–	0.891
Emergency	82 (7.9%)	62 (7.6%)	5 (20.8%)	7 (10.1%)	2 (3.3%)	7 (9.2%)	–	0.092
Abdominal wall	19 (1.7%)	15 (1.8%)	1 (4.2%)	2 (2.9%)	–	1 (1.3%)	–	0.878
Transplants	21 (2.0%)	16 (2.0%)	–	–	–	1 (1.3%)	–	0.924
Years of practice after graduation	10.7 ± 8.4 9.0 (IQR 11)	10.8 ± 8.3 9.0 (IQR 11)	11.5 ± 10.8 6.5 (15)	12.0 ± 10.1 11.0 (IQR 12)	6.8 ± 5.0 5.0 (IQR 7)	11.9 ± 8.3 10.9 (IQR 9)	12.5 ± 3.5 12.5 (IQR 2)	0.002
Practice level								
Trainee/fellow	247 (23.6%)	207 (25.2%)	4 (16.7%)	11 (15.9%)	9 (15.0%)	16 (21.1%)	2 (100%)	0.136
Consultant/attending	585 (55.9%)	452 (55.3%)	16 (66.6%)	40 (58.0%)	34 (56.7%)	46 (60.5%)	–	0.736
Researcher/Professor	177 (16.9%)	128 (15.7%)	4 (16.7%)	17 (24.6%)	16 (26.6%)	14 (18.4%)	–	0.094
Director	2 (0.2%)	1 (0.2%)	–	–	–	–	–	-
Other	35 (3.4%)	29 (3.6%)	–	1 (1.5%)	1 (1.7%)	–	–	0.657
Type of hospital								
Public (teaching)	794 (75.9%)	653 (80.0%)	11 (45.8%)	34 (49.3%)	48 (80.0%)	47 (61.9%)	2 (100%)	< 0.001
Private (teaching)	129 (12.3%)	76 (9.2%)	13 (54.2%)	25 (36.2%)	2 (3.3%)	14 (18.4%)	–	< 0.001
Public (no teaching)	90 (8.6%)	72 (8.8%)	–	4 (5.8%)	7 (11.7%)	7 (9.2%)	–	0.725
Private (no teaching)	33 (3.2%)	16 (2.0%)	–	6 (8.7%)	3 (5.0%)	8 (10.5%)	–	0.008
Hospital beds								
0–50	115 (11.0%)	106 (13.0%)	–	3 (4.3%)	5 (8.3%)	1 (1.3%)	–	0.015
51–100	90 (8.6%)	54 (6.6%)	1 (4.2%)	15 (21.7%)	12 (20.0%)	9 (11.8%)	–	< 0.001
101–200	116 (11.1%)	65 (8.0%)	2 (8.3%)	22 (31.9%)	14 (23.3%)	13 (17.2%)	–	< 0.001
201–400	227 (21.7%)	180 (22.0%)	5 (20.8%)	22 (31.9%)	9 (15.1%)	12 (15.8%)	–	0.116
> 400	498 (47.6%)	412 (50.4%)	16 (66.7%)	7 (10.2%)	20 (33.3%)	41 (53.9%)	2 (100%)	< 0.001
Private practice	417 (39.9%)	300 (36.8%)	5 (20.8%)	48 (69.6%)	33 (55.0%)	30 (39.5%)	–	< 0.001
Elective clinical practice besides on-call	1016 (97.2%)	794 (97.2%)	21 (87.5%)	68 (98.6%)	60 (100%)	74 (97.4%)	2 (100%)	0.064
N. on-calls/month	6.7 ± 4.9 5.0 (IQR 4)	6.5 ± 4.9 5.0 (IQR 4)	7.8 ± 4.1 7.0 (IQR 5)	7.0 ± 4.9 5.0 (IQR 4)	8.7 ± 6.1 7.0 (IQR 5)	6.3 ± 3.6 5.0 (IQR 4)	4.0 ± 1.4 4.0 (IQR 1)	0.005
% of on-call in presence								
0–25%	272 (26.0%)	191 (23.3%)	12 (50.0%)	22 (31.9%)	18 (30.0%)	30 (39.5%)	1 (50%)	< 0.001
26–50%	171 (16.4%)	127 (15.7%)	2 (8.3%)	8 (11.5%)	14 (23.4%)	18 (23.7%)	1 (50%)	0.082
51–75%	117 (11.1%)	96 (11.6%)	–	7 (10.1%)	7 (11.6%)	7 (9.2%)	–	0.769
76–100%	486 (46.5%)	403 (49.4%)	10 (41.7%)	32 (46.5%)	21 (35.0%)	21 (27.6%)	–	0.002
N. of hours of each on-call shift								
6–12	345 (33.0%)	299 (36.6%)	3 (12.5%)	15 (21.8%)	10 (16.7%)	18 (23.7%)	–	< 0.001
12–24	411 (39.3%)	307 (37.5%)	13 (54.2%)	32 (46.4%)	26 (43.3%)	33 (43.4%)	2 (100%)	0.227
18–24	134 (12.8%)	103 (12.6%)	2 (8.3%)	11 (15.9%)	11 (18.3%)	7 (9.2%)	–	0.464
25–36	100 (9.6%)	75 (9.2%)	3 (12.5%)	7 (10.1%)	2 (3.3%)	13 (17.1%)	–	0.027
37–48	15 (1.4%)	10 (1.2%)	–	1 (1.4%)	1 (1.7%)	3 (3.9%)	–	0.343
> 48	41 (3.9%)	23 (2.9%)	3 (12.5%)	3 (4.4%)	10 (16.7%)	2 (2.6%)	–	< 0.001
N. patients managed during an on-call	13.0 ± 15.7 10.0 (IQR 10)	12.5 ± 14.3 10.0 (IQR 10)	16.4 ± 13.1 15.0 (IQR 11)	14.4 ± 14.0 10.0 (IQR 15)	15.1 ± 28.9 8.0 (IQR 11)	14.6 ± 18.4 10.0 (IQR 14)	11.0 ± 1.4 11.0 (IQR 1)	0.390
N. weekend shifts on-call (or weekly public holidays)/month	1.7 ± 0.9 2.0 (IQR 1)	1.7 ± 0.8 2.0 (IQR 1)	2.1 ± 1.6 2.0 (IQR 1)	1.5 ± 0.9 2.0 (IQR 1)	2.3 ± 1.9 2.0 (IQR 1)	1.7 ± 0.9 2.0 (IQR 1)	1.0 ± 0.0 1.0 (IQR 0)	< 0.001
Patients from other specialties managed during the on-call shift	449 (42.9%)	355 (43.5%)	5 (20.8%)	26 (37.7%)	32 (53.3%)	31 (40.8%)	–	0.076
Involvement in research	944 (90.3%)	758 (92.8%)	21 (87.5%)	58 (84.1%)	43 (71.7%)	66 (86.5%)	2 (100%)	< 0.001
Involvement in teaching	885 (84.6%)	673 (82.4%)	24 (100%)	63 (91.3%)	55 (91.7%)	70 (92.1%)	2 (100%)	0.024
N. scientific articles published in scientific journals/year	6.1 ± 8.4 3.0 (IQR 7)	6.6 ± 8.6 3.0 (IQR 7)	5.4 ± 6.5 3.0 (IQR 3)	3.9 ± 6.6 2.0 (IQR 3)	3.2 ± 5.5 1.0 (IQR 2)	5.6 ± 9.9 2.0 (IQR 5)	13.5 ± 17.7 13.5 (IQR 12)	0.004
N. scientific articles read/month	12.2 ± 14.3 10.0 (IQR 10)	12.4 ± 14.4 10.0 (IQR 10)	15.3 ± 21.1 10.0 (IQR 12)	10.6 ± 10.2 9.0 (IQR 5)	3.2 ± 5.5 1.0 (IQR 2)	12.9 ± 15.9 6.0 (IQR 13)	4.0 ± 1.4 4.0 (IQR 1)	0.313
N. scientific conferences attended/year as faculty	2.8 ± 4.8 2.0 (IQR 2)	2.9 ± 5.1 2.0 (IQR 2)	2.9 ± 2.7 2.0 (IQR 1)	2.6 ± 2.7 2.0 (IQR 2)	1.2 ± 1.4 1.0 (IQR 2)	3.1 ± 4.7 2.0 (IQR 2)	1.0 ± 1.4 1.0 (IQR 1)	0.118
N. scientific conferences attended/year as learner	4.7 ± 6.0 3.0 (IQR 3)	4.5 ± 3.9 3.0 (IQR 3)	8.0 ± 24.0 3.0 (IQR 2)	5.9 ± 7.0 4.0 (IQR 3)	3.9 ± 5.5 2.0 (IQR 3)	5.2 ± 9.3 4.0 (IQR 3)	1.5 ± 0.7 1.5 (IQR 0.5)	0.013
Day-off after on-call shift	367 (35.1%)	327 (40.0%)	5 (20.8%)	14 (20.3%)	4 (6.7%)	18 (23.7%)	–	< 0.001
Two days off if on-call during weekly public holidays	109 (10.4%)	99 (12.2%)	2 (8.3%)	5 (7.2%)	–	2 (2.6%)	–	0.010

In Europe, 70.5% of responders were male, compared to 96.7% in Africa (p < 0.001). Age distribution data and years of practice showed variations underlining age-related demographic differences.

Hospitals with 0–50 beds were represented by 13% of responders in Europe and 1.3% in Asia (*p* = 0.015). Hospitals with > 400 beds were attended by 50.4% of European, 66.7% of North American, 53.9% of Asian, 33.3% of African, and 10.2% of South American responders (*p* < 0.001).

Data from the continent and professional level revealed significant differences in clinical workload, academic engagement, on-call activity, and post-on-call rest (Table 2).

**Table 2:** Study results stratified according to professional level (Trainee vs. Consultant/Professor) and Continent

			Overall	Europe	North America	South America	Africa	Asia	Oceania	p value
		Trainee	247 (27.0%)	207 (28.8%)	4 (16.7%)	11 (17.4%)	9 (15.0%)	16 (21.1%)	2 (100%)	
		Consultant/Professor	799 (73.0%)	610 (71.2%)	20 (83.3%)	58 (82.6%)	51 (85.0%)	60 (78.9%)	–	
Private practice		Trainee	32 (12.8%)	23 (11.3%)	–	3 (27.3%)	3 (30.0%)	3 (20.0%)	–	0.901* 0.151^a^
		Consultant/Professor	406 (50.8%)	276 (45.2%)	5 (25.0%)	45 (77.6%)	31 (60.0%)	27 (44.3%)	–	< 0.001^b^
			*p* value < *0.001*	*p* value < *0.001*	*p* value *0.814*	*p* value *0.008*	*p* value *0.125*	*p* value *0.056*	*p* value *NA*	
Elective clinical practice besides on-call		Trainee	236 (95.8%)	198 (95.9%)	4 (100%)	11 (100%)	9 (100.0%)	14 (86.7%)	2 (100%)	0.164* 0.252^a^
		Consultant/Professor	782 (97.9%)	595 (97.5%)	17 (85.0%)	57 (98.3%)	51 (100.0%)	60 (100.0%)	–	0.013^b^
			*p* value *0.047*	*p* value *0.164*	*p* value *0.785*	*p* value *0.210*	*p* value *0.185*	*p* value *0.045*	*p* value *NA*	
N. on-calls/month		Trainee	6.43 ± 5.20 5.0 (IQR 4)	6.38 ± 5.47 5.0 (IQR 3)	8.25 ± 2.38 8.5 (IQR 5.2)	5.27 ± 1.91 5.0 (IQR 3)	6.80 ± 3.86 7.0 (IQR 4)	7.66 ± 4.15 7.0 (IQR 5)	4.0 ± 1.0 4.0 (IQR 1.5)	0.078* 0.801^a^
		Consultant/Professor	6.61 ± 4.47 5.0 (IQR 4)	6.65 ± 4.60 5.0 (IQR 4)	7.70 ± 4.26 7.0 (IQR 5.7)	7.36 ± 5.16 5.0 (IQR 6)	9.18 ± 6.32 7.0 (IQR 6.5)	6.0 ± 3.35 5.0 (IQR 3.5)	–	0.001^b^
			*p* value *0.595*	*p* value *0.487*	*p* value *0.806*	*p* value *0.191*	*p* value *0.280*	*p* value *0.098*	*p* value *NA*	
% of on-call in presence	Trainee	0–25%	29 (11.7%)	25 (11.7%)	–	1 (9.1%)	2 (20.0%)	–	1 (50%)	0.426* 0.718^a^
	Consultant/Professor		259 (32.4%)	164 (26.3%)	12 (60.0%)	21 (36.2%)	17 (32.0%)	29 (49.2%)	–	< 0.001^b^
			*p* value < *0.001*	*p* value < *0.001*	*p* value *0.109*	*p* value *0.076*	*p* value *0.508*	*p* value *0.001*	*p* value *NA*	
	Trainee	26–50%	32 (12.8%)	21 (9.9%)	1 (25.0%)	2 (18.2%)	2 (20.0%)	4 (26.7%)	1 (50%)	0.745* 0.276^a^
	Consultant/Professor		136 (16.9%)	99 (16.0%)	1 (5.0%)	6 (10.3%)	12 (24.0%)	14 (23.0%)	–	0.116^b^
			*p* value *0.128*	*p* value *0.032*	*p* value *0.186*	*p* value *0.456*	*p* value *0.931*	*p* value *0.889*	*p* value *NA*	
	Trainee	51–75%	28 (11.3%)	26 (12.2%)	–	1 (9.1%)	–	2 (13.3%)	–	0.766* 0.951^a^
	Consultant/Professor		89 (11.2%)	76 (11.6%)	–	6 (10.3%)	7 (14.0%)	5 (8.2%)	–	0.714^b^
			*p* value *0.931*	*p* value *0.969*	*p* value *0.250*	*p* value *0.899*	*p* value *0.749*	*p* value *0.608*	*p* value *NA*	
	Trainee	76–100%	158 (64.2%)	135 (65.3%)	3 (75.0%)	7 (63.6%)	5 (60.0%)	10 (60.0%)	–	0.411* 0.965^a^
	Consultant/Professor		315 (39.5%)	271 (43.8%)	7 (35.0%)	25 (43.1%)	15 (30.0%)	12 (19.7%)	–	0.001^b^
			*p* value < *0.001*	*p* value < *0.001*	*p* value *0.138*	*p* value *0.210*	*p* value *0.125*	*p* value *0.008*	*p* value *NA*	
N. of hours of each on-call shift	Trainee	6–12	95 (38.5%)	92 (44.6%)	–	–	1 (10.0%)	3 (13.3%)	–	0.069* 0.013^a^
	Consultant/Professor		264 (33.1%)	195 (32.0%)	3 (15.0%)	15 (25.9%)	9 (18.0%)	16 (26.2%)	–	0.100^b^
			*p* value *0.116*	*p* value *0.046*	*p* value *0.785*	*p* value *0.226*	*p* value *0.627*	*p* value *0.515*	*p* value *NA*	
	Trainee	12–24	93 (37.7%)	75 (36.0%)	2 (50.0%)	4 (36.4%)	5 (60.0%)	2 (6.7%)	2 (100%)	0.161* 0.223^a^
	Consultant/Professor		305 (38.2%)	240 (39.5%)	11 (55.0%)	28 (48.3%)	20 (40.0%)	27 (45.9%)	–	0.400^b^
			*p* value *0.882*	*p* value *0.426*	*p* value *0.854*	*p* value *0.467*	*p* value *0.359*	*p* value *0.017*	*p* value *NA*	
	Trainee	18–24	21 (7.9%)	16 (7.7%)	–	5 (45.5%)	2 (20.0%)	2 (6.7%)	–	0.434* 0.001°
	Consultant/Professor		114 (14.3%)	91 (14.9%)	2 (10.0%)	10 (17.2%)	9 (18.0%)	6 (9.8%)	–	0.722^§^
			*p* value *0.018*	*p* value *0.008*	*p* value *0.538*	*p* value *0.037*	*p* value *0.743*	*p* value *0.772*	*p* value *NA*	
	Trainee	25–36	29 (12.1%)	18 (9.0%)	2 (50.0%)	1 (9.1%)	1 (10.0%)	4 (26.7%)	–	0.537* 0.027^a^
	Consultant/Professor		74 (9.1%)	54 (8.8%)	1 (5.0%)	2 (3.4%)	1 (2.0%)	9 (14.8%)	–	0.075^b^
			*p* value *0.253*	*p* value *0.945*	*p* value *0.012*	*p* value *0.400*	*p* value *0.185*	*p* value *0.345*	*p* value *NA*	
	Trainee	37–48	6 (2.6%)	4 (1.8%)	–	1 (9.1%)	–	3 (13.3%)	–	0.844* 0.001^a^
	Consultant/Professor		9 (1.1%)	6 (0.9%)	–	–	1 (2.0%)	1 (1.6%)	–	0.553^b^
			*p* value *0.132*	*p* value *0.283*	*p* value *0.250*	*p* value *0.181*	*p* value *0.191*	*p* value *0.006*	*p* value *NA*	
	Trainee	> 48	3 (1.1%)	2 (0.9%)	–	–	–	2 (6.7%)	–	0.075* < 0.001^a^
	Consultant/Professor		33 (4.2%)	24 (4.0%)	3 (15.0%)	3 (5.2%)	10 (20.0%)	1 (1.6%)	–	< 0.001^b^
			*p* value *0.026*	*p* value *0.035*	*p* value *0.785*	*p* value *0.610*	*p* value *0.469*	*p* value *0.048*	*p* value *NA*	
N. weekend shifts on-call (or weekly public holidays)/month	Trainee		1.75 ± 0.91 2.0 (IQR 1)	1.70 ± 0.86 2.0 (IQR 1)	2.50 ± 0.86 2.0 (IQR 1.5)	2.18 ± 1.11 2.0 (IQR 1)	1.60 ± 0.91 1.5 (IQR 1.2)	2.33 ± 1.07 2.0 (IQR 2)	1.0 ± 0.0 1.0 (IQR 0)	< 0.001* 0.008^a^
	Consultant/Professor		1.67 ± 1.00 2.0 (IQR 1)	1.69 ± 0.83 2.0 (IQR 1)	2.05 ± 1.68 2.0 (IQR 1)	1.44 ± 0.87 1.0 (IQR 1)	2.44 ± 1.98 2.0 (IQR 1.2)	1.62 ± 0.83 2.0 (IQR 1)	–	0.001^b^
			*p* value *0.262*	*p* value *0.882*	*p* value *0.611*	*p* value *0.015*	*p* value *0.218*	*p* value *0.005*	*p* value *NA*	
Involvement in research		Trainee	720 (90.1%)	563 (92.3%)	17 (85.0%)	50 (86.2%)	37 (72.0%)	51 (85.2%)	–	0.139* 0.028^a^
		Consultant/Professor	227 (91.8%)	191 (92.3%)	4 (100%)	8 (72.7%)	6 (70.0%)	14 (86.7%)	2 (100%)	< 0.001^b^
			*p* value *0.400*	*p* value *0.990*	*p* value *0.785*	*p* value *0.262*	*p* value *0.718*	*p* value *0.800*	*p* value *NA*	
Involvement in teaching		Trainee	182 (74.0%)	147 (71.2%)	4 (100%)	8 (72.7%)	9 (100.0%)	15 (93.3%)	2 (100%)	0.399* 0.236^a^
		Consultant/Professor	720 (90.1%)	520 (85.2%)	20 (100%)	55 (94.8%)	46 (90.0%)	55 (91.8%)	–	0.104^b^
			*p* value < *0.001*	*p* value < *0.001*	*p* value *0.250*	*p* value *0.017*	*p* value *0.984*	*p* value *0.783*	*p* value *NA*	
N. scientific articles published in scientific journals/year		Trainee	4.16 ± 7.15 2.0 (IQR 3)	4.04 ± 5.45 2.0 (IQR 4)	9.00 ± 9.45 5.5 (IQR 19.5)	1.72 ± 1.54 1.0 (IQR 3)	1.50 ± 1.28 1.0 (IQR 2)	7.20 ± 17.15 1.0 (IQR 2)	13.5 ± 12.5 13.5 (IQR 18.7)	< 0.001* 0.036^a^
		Consultant/Professor	7.71 ± 9.55 4.0 (IQR 8)	6.76 ± 8.41 3.5 (IQR 8)	4.70 ± 5.33 3.0 (IQR 3)	4.29 ± 7.09 2.5 (IQR 4)	3.54 ± 5.93 1.0 (IQR 3)	5.31 ± 7.03 2.0 (IQR 6)	–	0.001^b^
			*p* value < *0.001*	*p* value < *0.001*	*p* value *0.208*	*p* value *0.238*	*p* value *0.311*	*p* value *0.501*	*p* value *NA*	
N. scientific articles read/month		Trainee	11.10 ± 12.91 9 (IQR 7.5)	10.90 ± 12.88 10.0 (IQR 6)	9.75 ± 6.41 8.0 (IQR 13.7)	14.72 ± 17.06 10.0 (IQR 12)	14.16 ± 14.16 10.0 (IQR 8)	11.35 ± 9.87 5.5 (IQR 16.5)	4.0 ± 1.0 4.0 (IQR 1.5)	0.144* 0.837^a^
		Consultant/Professor	13.47 ± 15.45 10.0 (IQR 10)	12.16 ± 14.08 10.0 (IQR 10)	16.35 ± 22.26 10.0 (IQR 15.5)	9.77 ± 7.92 9.0 (IQR 5)	8.28 ± 9.40 5.0 (IQR 7)	13.27 ± 16.86 8.0 (IQR 13)	–	0.111^b^
			*p* value *0.029*	*p* value *0.256*	*p* value *0.568*	*p* value *0.130*	*p* value *0.115*	*p* value *0.665*	*p* value *NA*	
N. scientific conferences attended/year as faculty		Trainee	1.49 ± 1.82 1.0 (IQR 2)	1.43 ± 1.43 1.0 (IQR 2)	2.75 ± 2.16 2.5 (IQR 4.75)	0.90 ± 0.66 1.0 (IQR 1)	0.60 ± 0.91 0.6 (IQR 1)	3.0 ± 4.73 1.0 (IQR 2)	1.0 ± 1.0 1.0 (IQR 1.5)	< 0.001* 0.004^a^
		Consultant/Professor	3.69 ± 6.39 2.0 (IQR 3)	3.03 ± 4.48 2.0 (IQR 3)	2.90 ± 2.80 2.0 (IQR 1.75)	2.98 ± 2.83 2.0 (IQR 3)	1.32 ± 1.48 1.0 (IQR 2)	3.11 ± 4.68 2.0 (IQR 2)	–	0.098^b^
			*p* value < *0.001*	*p* value < *0.001*	*p* value *0.920*	*p* value *0.018*	*p* value *0.164*	*p* value *0.933*	*p* value *NA*	
N. scientific conferences attended/year as learner		Trainee	4.56 ± 5.84 3.0 (IQR 3)	4.23 ± 3.31 3.0 (IQR 3)	2.25 ± 1.29 2.0 (IQR 2.7)	5.09 ± 4.94 4.0 (IQR 3)	6.70 ± 7.07 4.0 (IQR 8.7)	8.8 ± 19.08 4.0 (IQR 3)	1.5 ± 0.5 1.5 (IQR 0.7)	0.197* 0.043^a^
		Consultant/Professor	4.82 ± 5.83 3.0 (IQR 3)	4.53 ± 4.11 3.0 (IQR 3)	9.20 ± 25.54 3.0 (IQR 4.2)	6.13 ± 7.29 4.0 (IQR 3.7)	3.44 ± 4.92 2.0 (IQR 3)	4.39 ± 3.79 3.0 (IQR 3)	–	0.001^b^
			*p* value *0.540*	*p* value *0.342*	*p* value *0.598*	*p* value *0.652*	*p* value *0.092*	*p* value *0.093*	*p* value *NA*	
Day–off after on-call shift	Trainee		100 (40.8%)	91 (44.1%)	2 (50%)	2 (18.2%)	1 (10.0%)	5 (33.3%)	–	0.796* 0.125^a^
	Consultant/Professor		269 (33.7%)	234 (38.3%)	3 (15.0%)	12 (20.7%)	3 (6.0%)	13 (21.3%)	–	< 0.001^b^
			*p* value *0.049*	*p* value *0.154*	*p* value *0.115*	*p* value *0.849*	*p* value *0.562*	*p* value *0.423*	*p* value *NA*	
Two days off if on-call during weekly public holidays	Trainee		28 (11.3%)	27 (13.1%)	1 (25.0%)	–	–	–	–	0.874* 0.856^a^
	Consultant/Professor		78 (9.8%)	74 (12.1%)	1 (5.0%)	5 (8.6%)	–	2 (3.3%)	–	0.042^b^
			*p* value *0.473*	*p* value *0.730*	*p* value *0.186*	*p* value *0.959*	*p* value *0.185*	*p* value *0.594*	*p* value *NA*	

*General difference among Trainees and Consultants/Professors in the different Continents

^a^Difference among Trainees in the different Continents

^b^Difference among Consultants/Professors in the different Continents

### Clinical workload

**Fig. 2:**
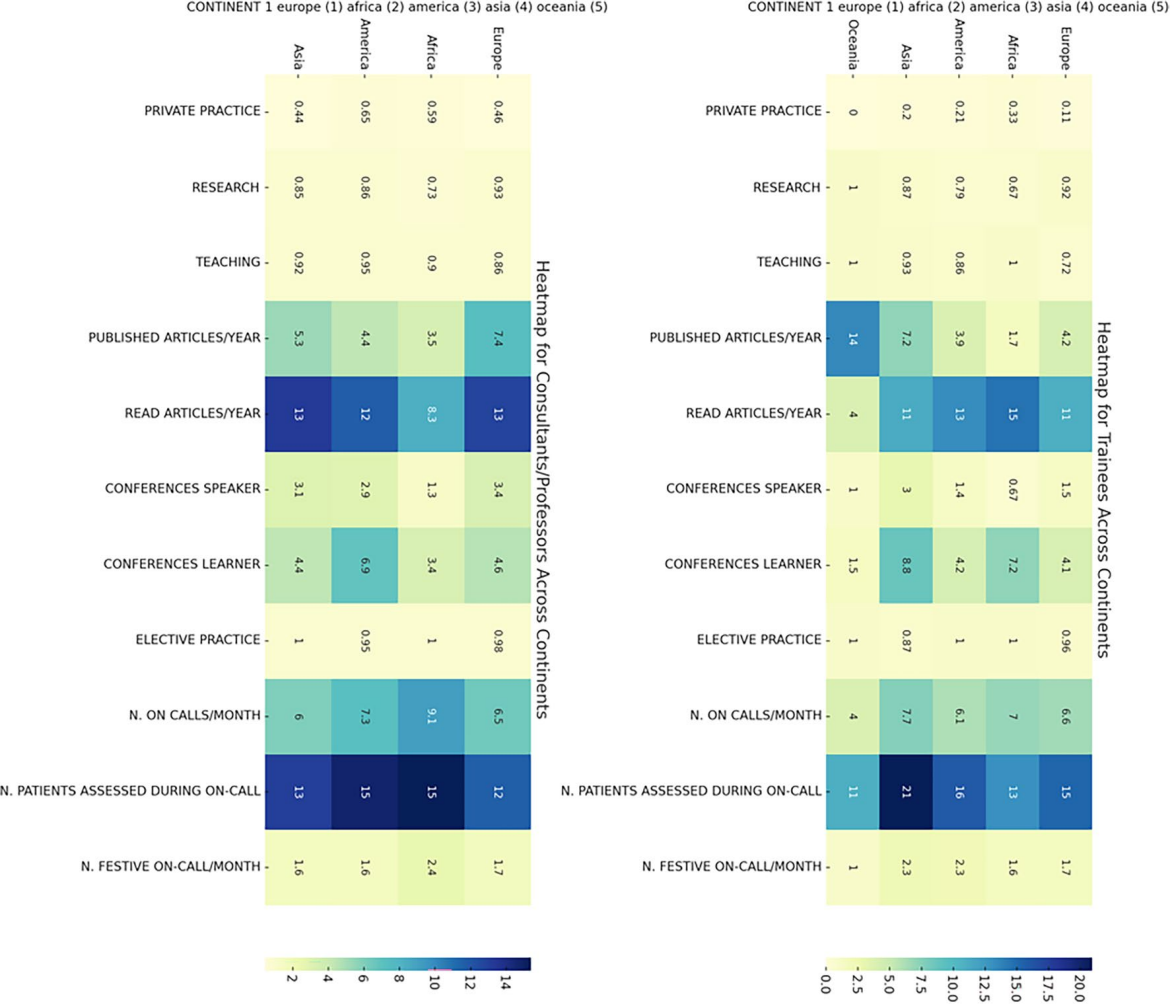
Distribution of clinical and academic responsibilities across continents (Consultant/Professor Left, Trainee Right). This heatmap illustrates the comparison of clinical and academic tasks undertaken by medical trainees and consultants across five continents. Color intensity correlates with the frequency of each activity, ranging from private practice to on-call duties, with darker shades indicating higher prevalence

**Fig. 3:**
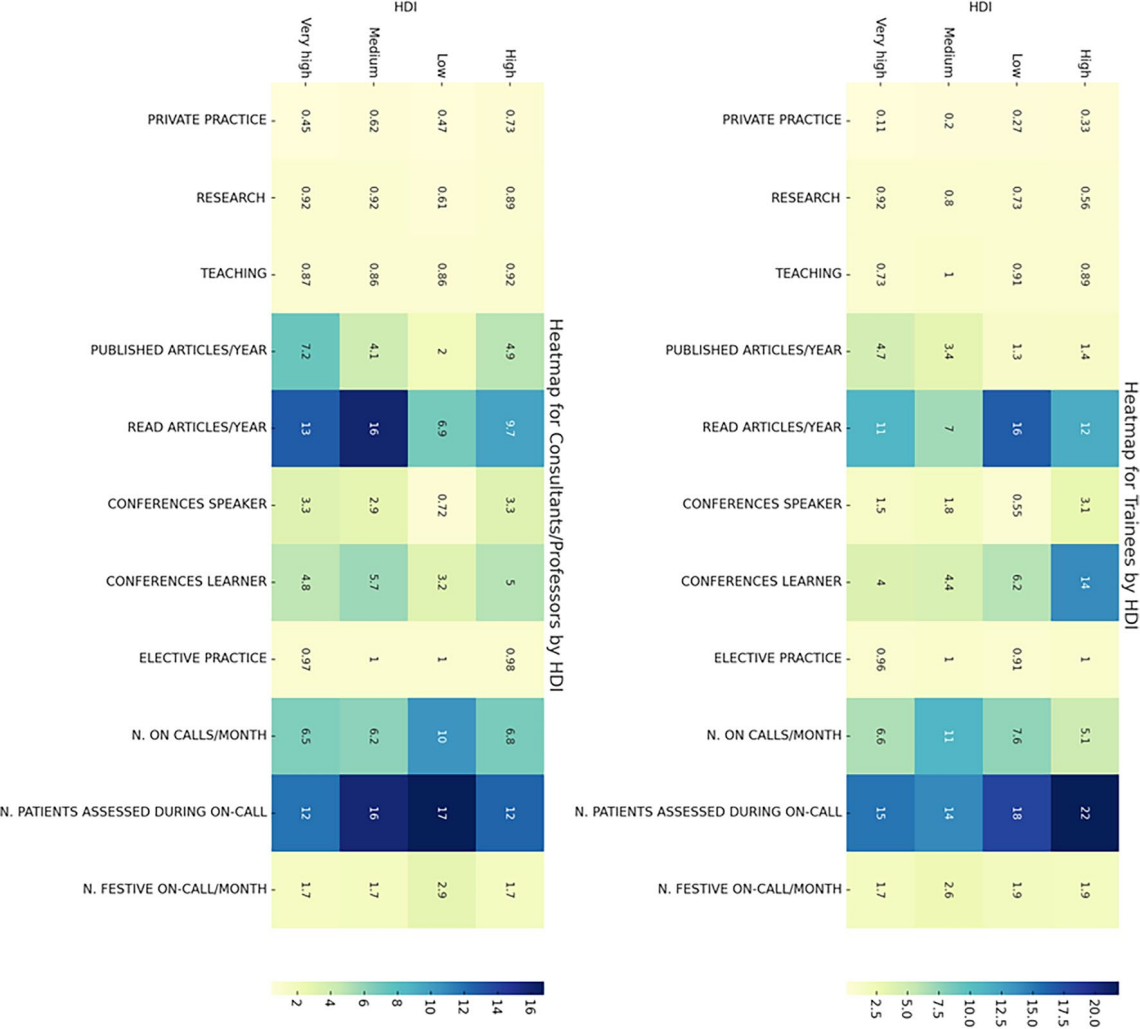
Distribution of clinical and academic responsibilities by Human Development Index (HDI) (Consultant/Professor Left, Trainee Right). This heatmap displays the variance in clinical and academic responsibilities among medical trainees and consultants/professors across countries with different HDI levels. Color intensity correlates with the frequency of each activity, ranging from private practice to on-call duties, with darker shades indicating higher prevalence

The distributions of clinical and academic responsibilities across continents and across different HDI levels are reported in the Figs. 2 and 3.

In Europe, 80% of responders worked in public teaching hospitals vs 45.8% in North America (*p* < 0.001). In North America 54.2% worked in private teaching hospitals vs 9.2% in Europe (*p* < 0.001); 39.9% of respondents declared that they had a private practice in addition to their ordinary activity. The survey data on private practice across continents showed South America and Africa at the top, with 69.6% and 55.0% of responders engaged in private practice, followed by Asia (39.5%) and Europe (36.8%) (*p* < 0.001).

Twelve-point eight percent of trainees were involved in private practice compared to 50.8% consultants/professors. This disparity was consistent across continents (11.3% vs 45.2% in Europe, 27.3% vs 77.6% in South America, 30% vs 60% in Africa).

### Academic engagement

The survey data on research activity showed overall high engagement across continents, with significant disparities. In Europe 92.8% of responders were involved in research, 87.5% in North America, and 71.7% in Africa (*p* < 0.001).

The mean number of research articles published in indexed journals in a year was 6.1(± 8.4), and the mean number of articles read monthly was 12.1(± 14.3). The number of scientific articles published yearly varied (*p* = 0.004), with responders in Europe (6.6 ± 8.6) publishing the most. The number of articles published by European consultants/professors was 6.76(± 8.41) versus 9.00(± 9.45) by North American trainees.

Involvement in teaching was 100% for North American and 91.7% for African responders, compared to 82.4% for Europeans (*p* = 0.024).

The mean number of conferences attended as a speaker in a year was 2.8(± 4.8), and the mean number of conferences attended as a learner was 4.7(± 6). In Europe, a mean of 4.5(± 3.9) conferences per year was attended, 8(± 24) in North America, 5.9(± 7) in South America, 5.2(± 9.3) in Asia, and 3.9(± 5.5) in Africa (p = 0.013). For African trainees, the mean was 0.60(± 0.91) conferences attended as faculty, compared to 0.90(± 0.66) for South American trainees; the same occurred with consultants/professors in these areas (1.32 ± 1.48 and 2.98 ± 2.83 conferences).

### On-call activity and post-on-call rest

The mean number of on-call shifts per month was 6.7(± 4.9), of which 46.5% in 76–100% cases in-house. The duration of the on-call shift was 12–24 h in 39.3% of cases, 6–12 h in 33% of cases, 18–24 h in 12.8% of cases, 25–36 h in 9.6% of cases, 37–48 h in 1.4% of cases and more than 48 h in 3.9% of cases.

European responders had a mean of 6.5(± 4.9) on-calls per month, with 49.4% requiring in-house presence in 76–100% of the cases, compared with 7.8(± 4.1, 41.7% requiring in-house presence in 76–100% of the cases) in North America, and 8.7(± 6.1, 35.0% requiring in-house presence in 76–100% of the cases) in Africa (*p* = 0.005). During an on-call shift, the mean number of patients assessed was 13(± 15.7), and 42.9% of responders stated they usually manage patients outside their specialty. The number of on-calls per month for trainees and consultants/professors across continents showed significant variability. In Europe, trainees had 6.38(± 5.47) on-calls, while consultants/professors had 6.65(± 4.60); in North America 8.25(± 2.38) vs 7.70(± 4.26) of consultants/professors; in Asia, 7.66(± 4.15) vs 6.0(± 3.35); in Africa 6.80(± 3.86) vs 9.18(± 6.32), and 5.27(± 1.91) vs 7.36(± 5.16) in South America. Consultants/professors, when on-call, were less likely to be required to stay in the hospital compared to trainees. This pattern was observed across various regions. Regarding on-call shift hours exceeding 48, 0.9% of trainees and 4.0% of consultants/professors in Europe had such shifts, compared with 20% of consultants/professors in Africa and 6.7% of trainees in Asia. In North America, no trainees and 15% of consultants/professors experienced > 48-h shifts.

**Fig. 4:**
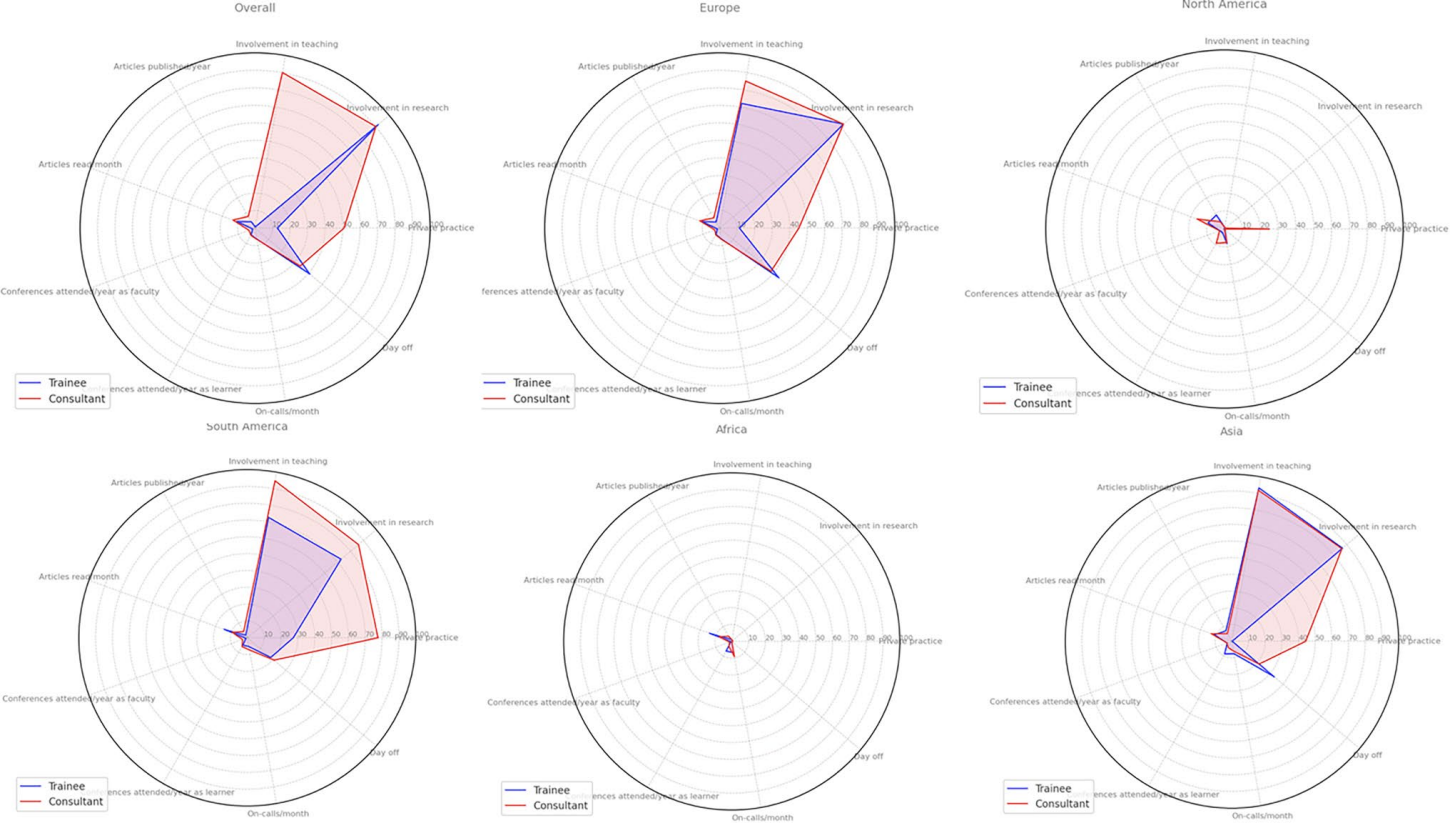
Professional activity profiles by continent and role. This radar chart compares the distribution of clinical and academic tasks among medical trainees and consultants/professors across four different continents: Europe, North America, South America, and Asia. Each colored shape represents the composite profile for trainees (blue) and consultants/professors (red) within each continent, across activities like teaching, research activities, private practice and clinical duties. The extent of each axis reflects the level of engagement in the corresponding activity

Overall, 35.1% of respondents declared having a day off after an on-call shift, while 10.4% declared having two days off in the week in which they were on-call during a public holiday. The data on days off after on-call shifts showed significant regional differences. In Europe, 40% of respondents got a day off after an on-call shift vs 20.8% in North America, 20.3% in South America, 23.7% in Asia, and 6.7% in Africa (*p* < 0.001). For trainees, 40.8% reported having a day off after an on-call shift, with variations by continent. Consultants/professors had a lower overall percentage of 33.7% receiving a day off after on-call shifts. In Europe, 44.1% of trainees received a day off vs 38.3% of consultants/professors, in North America 50% vs 15%, in Africa, 10% vs 6%, in Asia 33.3% vs 21.3%, and in South America 18.2% vs 20.7%. The different professional activity profiles by continent are reported with radar charts in Fig. 4.

### Analysis of predictive factors of day-off after on-call

**Fig. 5:**
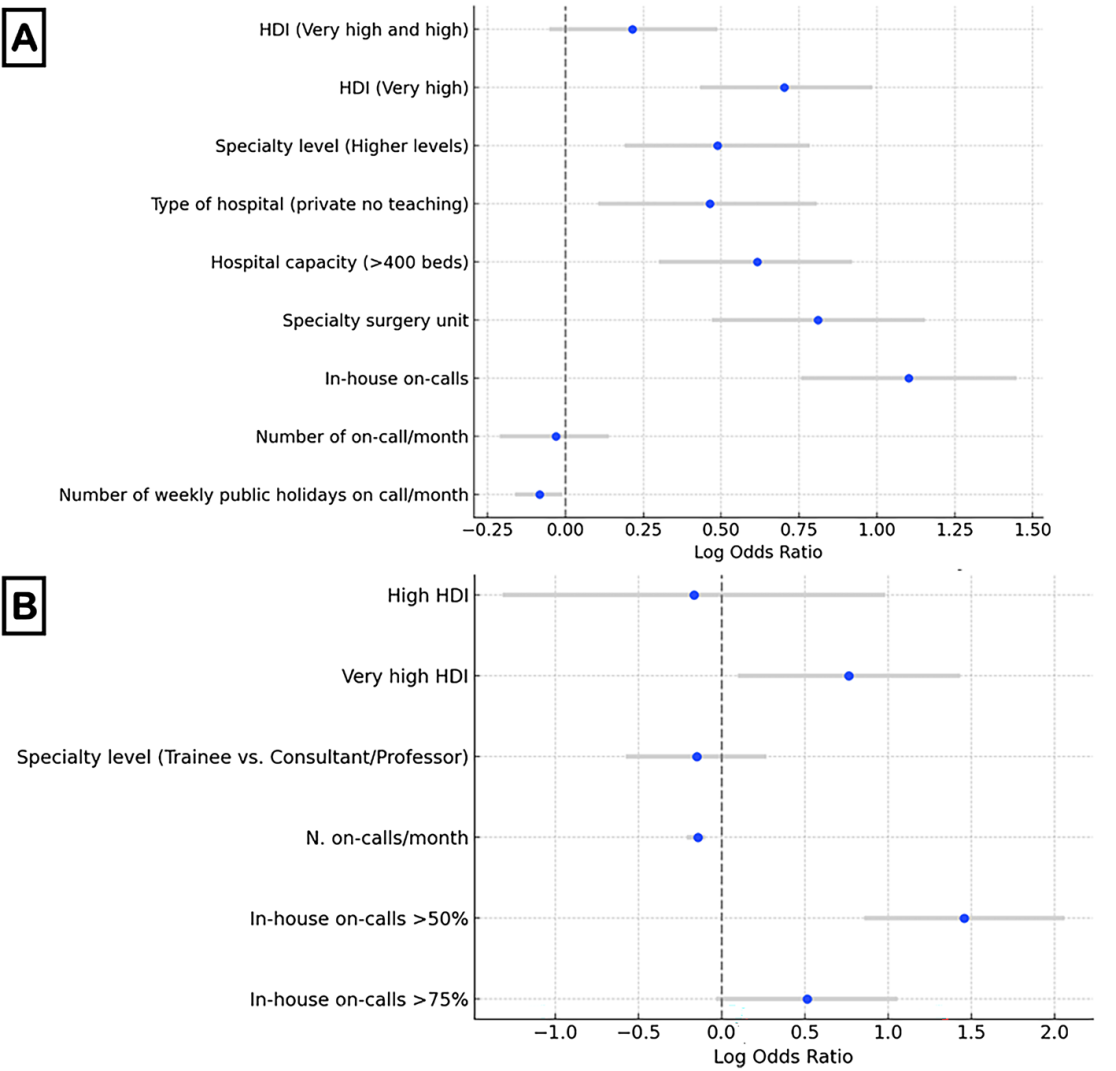
Independent predictors of a day-off following on-call duties—Multivariable Analysis. General population of responders (**A**); Subgroup analysis of responders who perform > 12 h on-calls (**B**)

In the univariable analysis of predictive factors for having a day off after on-call, both very high and high HDI (OR 3.131) and very high HDI (OR 2.542), hospital capacity over 400 beds (OR 3.007), working in a specialty surgery unit (OR 2.389), and doing in-house on-calls for 76–100% of the cases (OR 5.355) were associated with a day off. Working as consultants (OR 0.678), in private non-teaching hospitals (OR 0.358), and high numbers of weekly public holidays on-call per month (OR 0.848) were associated with the lack of a day off after on-call (Supplementary Material Table 2). Continents with a higher mean number of on-calls per month showed a more significant percentage of surgeons receiving a day off afterward, but this was not uniform across all groups or regions (Supplementary Material Fig. 1). There was an overall positive correlation between the duration of on-call shifts and the likelihood of receiving a subsequent day off. Longer on-call shifts tended to be associated with a higher probability of having a rest day post-on-call across various continents and roles (Supplementary Material Fig. 1). On the general adjusted multivariable analysis HDI (aOR 1.993), hospital capacity > 400 beds (aOR 2.423), working in a specialty surgery unit (aOR 2.087), and in-house on-call (aOR 5.446), predicted the likelihood of having a day off after an on-call shift (Supplementary Material Table 3). Working as consultant/professor (aOR 0.713), and having a high number of on-calls per month (aOR 0.917) were risk factors for not having a day off after on-call. When looking at independent predictors of a day-off following on-call duties in the subgroup of responders who performed extended on-call shifts > 12 h (Supplementary Material Table 4), a Very High HDI (aOR 2.148) and doing in-house on-calls in > 50% of the cases (aOR 4.297) were associated with the chance of having a day-off. Independent predictors of a day off following on-call duties are depicted in Fig. 5.

## Discussion

The LIONESS study provided the first comparative overview of the engagement levels of surgical trainees and consultants/professors across various professional domains on different continents. The current study showed that access to scientific research, opportunities for professional development, and the clinical workload in terms of on-calls, length of shifts, and the possibility of having 24 h free to rest afterwards differ depending on the professional level, the continent, and the HDI of the countries.

Alarmingly, 1.1% of trainees and 4.2% of consultants who took the current survey still carried out on-call shifts lasting more than 48 h. In Europe, where the law imposes stringent rules on the length of work shifts, 4% of consultants and 0.9% of trainees stated that they still carried out on-call shifts > 48 h. These percentages were even higher in South America (5.2%), North America (15%) and Africa (20%) among consultants, while in Asia, 6.7% of trainees worked shifts > 48 h. The effects of sleep deprivation on surgeons have been widely investigated, and many studies have reported its negative impact on cognition, attention, and performance [10, 15]. In consideration of the research in this field, the US Accreditation Council for Graduate Medicine Education (ACGME) Work Group on Resident Duty Hours first reform, in 2003, established a maximum 80-h work week and prohibited shift lengths from exceeding 30 consecutive hours [5]. The second reform capped shift lengths at 16 consecutive hours for interns and 28 h for other trainees [16]. The European Working Time Directive for physicians is even stricter, with an average limit of 48 h a week, 11 h of continuous rest every 24 h, a day off each week, a 20-min rest break every six hours, and a limit of eight hours worked in every 24 h for night work [11].

A relevant finding of our study was that in the general analysis, 40.8% of trainees and 33.7% of consultants/professors could not have a 24-h rest after an on-call shift, which is now considered a fundamental recovery practice. The distribution of this variable showed differences based on professional levels and different continents. As far as consultants were concerned, only 6% of those working in Africa could benefit from a 24-h rest after an on-call shift, compared to 38.3% of European consultants. For trainees, more than 40% of those working in Europe and North America were likely to have a day of rest after the on-call shift, compared to only 10% in Africa and 18.2% in South America. How fatigue affects surgeon performance remains an intriguing field of research. Interestingly, Yaghoubian et al*.* [17] showed that trauma surgery performed at night by residents who have worked longer than 16 h have similar favorable outcomes compared with those performed during the day. Conversely, Dinges et al*.* reported that continuous wakefulness of periods of more than 21 h is a major predictor of performance errors [18]. It is difficult to establish a specific correlation between the number of hours worked when proceeding with surgery and potential adverse outcomes, as these may be affected by numerous other variables not related to the length of the surgeon's working shift. Moreover, potential correlations may be limited by insufficient power to detect such associations reliably. However, the findings from previous studies suggested that for surgeons during night shift floats, protected days off work are fundamental [16, 19–21]. Dunn et al*.* [22] showed that a three-day period of recovery is insufficient for restorative sleep, while another study by Brown et al*.* [23] concluded that recovery of sleep duration, efficiency, and quality necessitate five full days. In the current study, the possibility of having adequate rest after an on-call shift of 24 h was shown to be associated with working in a country with a Very High HDI (aOR 1.993), working in large hospitals with > 400 beds (aOR 2.423), in a specialist surgery unit (aOR 2.087) and carrying out in-house on-call shifts (aOR 5.446). It should be noted that holding more experienced roles, such as consultants, was associated with the probability of being unable to take advantage of the day off after an on-call shift.

HDI has been used for comparing surgical outcomes, access to surgery, surgical techniques across countries with different HDI scores [24], and resource allocation understanding health disparities [25], but there is not enough evidence to directly establish a correlation between HDI and surgeon workload and on-call shifts. The LIONESS study revealed a groundbreaking association between HDI and surgeons' likelihood of having a day off following on-call duties. In regions with higher HDI, surgeons were more likely to benefit from rest periods post-on-call. This finding is significant, as it links HDI not only to economic and educational outcomes but also to working conditions in healthcare. This correlation underscores the need for policies prioritizing healthcare worker well-being, especially in lower HDI regions.

For trainees, there appeared to be a high degree of variability across continents in categories such as research and conference attendance, suggesting differences in training focus or career opportunities availability. For consultants/professors, the engagement levels across these categories were more evenly distributed.

Similarly, although research activity was high among the participants in this study in terms of involvement in research activities, teaching, number of scientific articles published and read, and number of scientific conferences attended as speakers and learners, essential differences between continents were observed. Involvement in teaching was relatively higher among trainees in Africa compared to other continents. In contrast, trainees in Europe and North America tended to participate more in research than their counterparts in South America and Africa. In particular, research activity was less accessible in Africa than in other continents, especially for surgeons in training. There was a marked difference in the number of scientific articles published yearly. This varied significantly across continents, with Europe and North America leading. The mean number of scientific articles published yearly showed a drastic reduction in South America and Africa compared to other continents. It should also be noted that, in general, surgeons with top roles published more scientific articles than surgeons in training, thus outlining a probable lack of research training in younger groups who should instead constitute the driving force of scientific research.

Strategic recommendations can be proposed to balance surgeon workloads, enhance research opportunities, and ensure adequate rest. Our analysis suggests that healthcare policies and practices must be tailored to the unique challenges and resources of each region. We advise implementing a system that allows for flexibility within a standardized framework, accommodating the specific needs of different healthcare settings and ensuring surgeons have predictable and manageable workloads. In lower HDI countries, we suggest focusing on optimizing resource allocation by introducing telemedicine consultations to reduce the unnecessary physical workload on surgeons and by utilizing mid-level practitioners, such as nurse practitioners or physician assistants, to handle non-surgical or basic surgical tasks [26]. Additionally, establishing global research collaboratives that include surgeons from high, medium, and low HDI countries is crucial to ensure diverse participation and access to research opportunities, fostering an environment of shared learning and development [27]. Dedicated funding streams for research initiatives specifically designed for lower HDI regions could promote local research development and publication opportunities. For higher HDI regions, adopting legislation that mandates minimum rest periods for surgeons, similar to regulations in place for other high-stress professions, is critical. A potential strategy could be to implement systems that allow for shift swapping and provide additional support during peak times, ensuring that no single individual is overwhelmed, which can be particularly beneficial in settings with limited staffing. Moreover, leveraging digital tools and artificial intelligence for scheduling, administrative tasks, and even preliminary diagnostics can reduce the workload on surgeons, allowing them to focus on essential surgical duties and research.

### Strengths and limitations

The main strengths of the LIONESS study are the employment of a standard survey methodology, the large sample size, and the inclusion of all continents, offering a holistic view of surgeons' professional engagements.

A notable limitation of this study is the significant gender disparity among the respondents, with a predominance of male participants. This imbalance reflects a broader issue within the surgical field but also limits the applicability of our findings across genders. The perspectives and experiences of female surgeons, who may face unique challenges and work-life balance issues in various healthcare systems, are underrepresented in our analysis. This gender imbalance could potentially skew the perception of workloads, rest periods, and opportunities for academic engagement. Future research should strive for a more balanced gender representation to capture a comprehensive view of the surgical profession's dynamics. Although efforts were made to encourage global recruitment across all HDI countries, participation from Very High HDI countries was more substantial, potentially introducing a selection bias among collaborating institutions. Last, most of the respondents (65.1%) were from general surgery units, while 34.9% were from subspecialty surgery units. This distribution could influence the survey findings, as experiences and practices might differ between general surgeons and those in surgical subspecialties. The predominance of participants from teaching hospitals likely influenced the responses related to academic activities, such as the number of articles published and read. Surgeons affiliated with academic institutions often have greater exposure to and encouragement for engaging in research activities as part of their professional roles. As a result, their reported levels of research activity and engagement with scientific literature could be higher than those of their counterparts in non-teaching hospitals. In these settings, the focus may be more heavily weighted towards clinical duties, with fewer resources and incentives for research available. Therefore, the experiences and challenges of surgeons in non-teaching, community, and rural hospitals, where academic and research activities might be less emphasized or supported, could significantly diverge from our study's findings. Efforts to engage a broader and more diverse participant pool in future studies could include targeted outreach to non-teaching hospitals and the incorporation of questions specifically designed to unearth the unique challenges and workloads faced by surgeons in these environments.

## Conclusions

The LIONESS study revealed critical insights into the disparities in workload, access to research, and professional opportunities for surgeons across different continents. HDI was associated with the likelihood of surgeons having a day off post-on-call, with higher HDI regions offering better work-life balance. Despite regulatory rules introduced in several national healthcare systems, many surgeons continue to work long shifts without adequate rest. Finally, the study highlighted uneven access to research and scientific meetings, further influenced by geographic and possibly economic factors. These findings call for global initiatives to promote equality in healthcare work environments and professional development opportunities.

## Supplementary Information

Below is the link to the electronic supplementary material.

Supplementary file1 Figure 1. Correlation between on-call frequency and post-call rest (A). This figure presents the relationship between the average number of on-call shifts per month (x-axis) and the corresponding percentage of medical staff receiving a day off after on-call duties (y-axis), stratified by trainees (circles) and consultants/professors (squares) across different continents. Association between on-call shift duration and post-call rest days (B). This scatter plot illustrates the relationship between the average duration of on-call shifts in hours (x-axis) and the percentage of medical staff receiving a day off after on-call duties (y-axis). Data points are distinguished between trainees (circles) and consultants/professors (squares), across different continents. (TIFF 4538 KB)

Supplementary file2 Table 1. Structure of the survey questionnaire. (DOC 41 KB)

Supplementary file3 Table 2. Results of the univariable analysis of predictive factors of day-off after on-call (General population of responders). (DOC 22 KB)

Supplementary file4 Table 3. Results of the multivariable analysis of predictive factors of day-off after on-call (General population of responders). (DOC 15 KB)

Supplementary file5 Table 4. Results of the multivariable analysis of predictive factors of day-off after on-call (Population of responders who perform extended on-calls > 12 h). (DOC 14 KB)

## Data Availability

All information is freely available by application to the Chief Investigator Mauro Podda (Department of Surgical Science, University of Cagliari).

## Declarations

### Privacy

The LIONESS data analysis process was initially conducted anonymously to ensure privacy and confidentiality. However, in recognition of the contributions of survey participants, the names of respondents were subsequently included for co-authorship. This approach was carefully balanced to maintain the data's integrity while acknowledging participants' valuable input. Including names for co-authorship was conducted with the explicit consent of the respondents, ensuring that their participation and acknowledgment aligned with ethical research practices.
