# Supplementary figures and images for: Global disparities in surgeons’ workloads, academic engagement and rest periods: the on-calL shIft fOr geNEral SurgeonS (LIONESS) study
Source: Updates Surg. 2024 Apr 29;76(5):1615–33. doi: 10.1007/s13304-024-01859-7 (PMC11455666; doi:10.1007/s13304-024-01859-7)

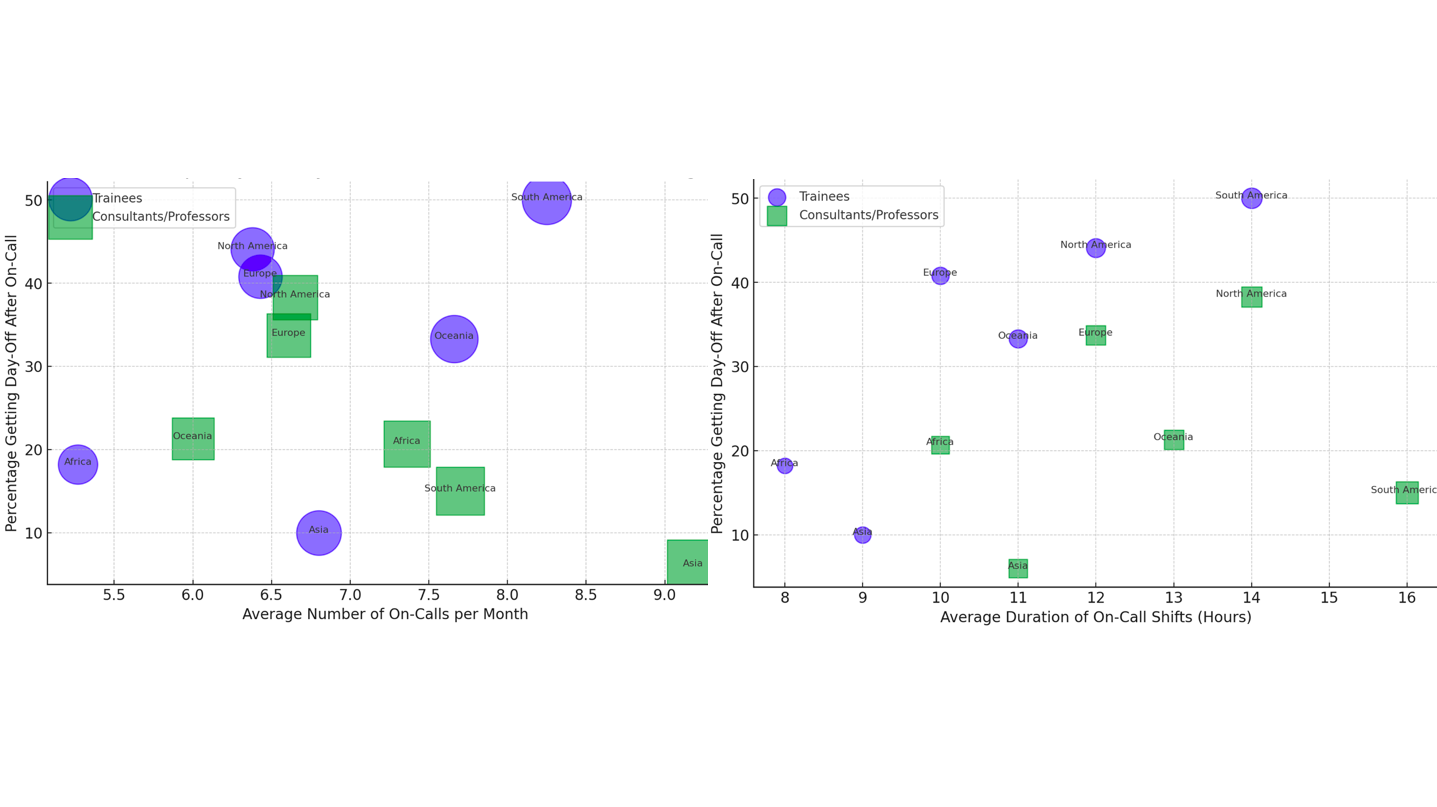

Supplement: Supplementary file 1 — Supplementary file1 Figure 1. Correlation between on-call frequency and post-call rest (A). This figure presents the relationship between the average number of on-call shifts per month (x-axis) and the corresponding percentage of medical staff receiving a day off after on-call duties (y-axis), stratified by trainees (circles) and consultants/professors (squares) across different continents. Association between on-call shift duration and post-call rest days (B). This scatter plot illustrates the relationship between the average duration of on-call shifts in hours (x-axis) and the percentage of medical staff receiving a day off after on-call duties (y-axis). Data points are distinguished between trainees (circles) and consultants/professors (squares), across different continents. (TIFF 4538 KB) [file 13304_2024_1859_MOESM1_ESM.tiff]
