# Supplementary material for: Global disparities in surgeons’ workloads, academic engagement and rest periods: the on-calL shIft fOr geNEral SurgeonS (LIONESS) study
Source: Updates Surg. 2024 Apr 29;76(5):1615–33. doi: 10.1007/s13304-024-01859-7 (PMC11455666; doi:10.1007/s13304-024-01859-7)
Supplement: Supplementary file 2 — Supplementary file2 Table 1. Structure of the survey questionnaire. (DOC 41 KB) [file 13304_2024_1859_MOESM2_ESM.doc]

**Supplementary Material Table 1.** Structure of the survey questionnaire

| **Section 1. Baseline information of the participants** | | | |
| --- | --- | --- | --- |
| **Question** | **Possible answers** |  |  |
| **Name** | Short answer (open) |  |  |
| **Surname** | Short answer (open) |  |  |
| **Sex** | Multiple choice | Man  Woman |  |
| **Age** | Short answer (open) |  |  |
| **In which Country are you practicing?** | Dropdown list | All Countries |  |
| **In which Continent are you practicing?** | Dropdown list | Europe  North America  South America  Africa  Asia  Oceania |  |
| **Name of your hospital** | Short answer (open) |  |  |
| **City** | Short answer (open) |  |  |
| **ORCID number** | Short answer (open) |  |  |
| **Type of sub-specialty** | Multiple choice | UpperGI  Colorectal  HPB  Emergency  Breast  Abdominal wall  Endocrine  Transplants  General surgery |  |
| **Years of practice (after graduation)** | Short answer (open) |  |  |
| **Professional level** | Multiple choice | Trainee/fellow  Consultant/attending  Professor/researcher |  |
|  |  |  |  |
| **Section 2. Hospital organization** | | | |
| **Question** | **Possible answers** |  |  |
| **Type of hospital** | Multiple choice | Public (teaching)  Public (no teaching)  Private (teaching)  Private (no teaching) |  |
| **Number of hospital beds** | Multiple choice | 0-50  51-100  101-200  201-400  More than 400 |  |
| **Do you work in a subspecialty unit (e.g. endocrine, Upper GI, etc) or in a general surgery Unit (more then one specialty present)?** | Multiple choice | Subspecialty unit  General surgery unit |  |
|  |  |  |  |
| **Section 3. Information about surgeon practice** | | | |
| **Question** | **Possible answers** |  |  |
| **Apart from your main clinical practice do you have a private practice?** | Multiple choice | Yes  No |  |
| **Are you usually involved in research projects?** | Multiple choice | Yes  No |  |
| **Apart from your main clinical practice, are you involved in teaching (students and/or trainees)?** | Multiple choice | Yes  No |  |
| **How many scientific articles in indexed journals do you publish/year?** | Short answer (open) |  |  |
| **How many scientific articles do you read per month?** | Short answer (open) |  |  |
| **How many conferences do you attend as speaker per year?** | Short answer (open) |  |  |
| **How many conferences do you attend as learner per year?** | Short answer (open) |  |  |
|  |  |  |  |
| **Section 4. On-call shift evaluation** | | | |
| **Question** | **Possible answers** |  |  |
| **Beside on-call surgery, are you doing regular clinical surgical practice in your hospital?** | Multiple choice | Yes  No |  |
| **How many on-calls do you perform per month (average) ?** | Short answer (open) |  |  |
| **In which percentage they are in presence (you NEED to stay in the hospital for all on-call shift)?** | Multiple choice | 0-25%  26-50%  51-75%  76-100% |  |
| **How long does an on-call shift usually last?** | Multiple choice | 6-12 hours  12-24 hours  25-36 hours  37-48 hours  More than 48 hours |  |
| **How many patients do you see (average) during an on-call shift?** | Short answer (open) |  |  |
| **How many week holiday shifts do your perform per month?** | Short answer (open) |  |  |
| **After an on-call shift do you have a day off?** | Multiple choice | Yes  No |  |
| **If you are on-call during public holiday do you have 2 days off?** | Multiple choice | Yes  No |  |
| **During the on-call shift do you manage patients from other specialties (urology/ENT/ortho, etc)?** | Multiple choice | Yes  No |  |
|  |  |  |  |
| **Section 5. Consent to participate** | | | |
| **Question** | **Possible answers** |  |  |
| **I would like to be contacted by the Authors for study updates and proposal.** | Multiple choice | Yes  No |  |
| **I hereby consent to the processing of the personal data that I have provided for the LIONESS ACIE study to get the authorship.** | Multiple choice | Yes  No |  |
|  |  |  |  |
| **Section 6. Comments** | | | |
| **Question** | **Possible answers** |  |  |
| **If you have any comments or you wants to add some additional information about the answers of this survey, please include these in this box.** | Short answer (open) |  |  |
