# Supplementary material for: Global disparities in surgeons’ workloads, academic engagement and rest periods: the on-calL shIft fOr geNEral SurgeonS (LIONESS) study
Source: Updates Surg. 2024 Apr 29;76(5):1615–33. doi: 10.1007/s13304-024-01859-7 (PMC11455666; doi:10.1007/s13304-024-01859-7)
Supplement: Supplementary file 3 — Supplementary file3 Table 2. Results of the univariable analysis of predictive factors of day-off after on-call (General population of responders). (DOC 22 KB) [file 13304_2024_1859_MOESM3_ESM.doc]

**Supplementary Material Table 2.** Results of the univariable analysis of predictive factors of day-off after on-call.

| **Variable** | **Odds Ratio** | **95% Confidence Interval** | **P value** |
| --- | --- | --- | --- |
| Sex | 1.272 | 0.954;1.697 | 0.101 |
| Age | 0.992 | 0.977;1.010 | 0.331 |
| HDI* (Very high and high) | 3.131 | 1.793;5.623 | <0.001 |
| HDI* (Very high) | 2.542 | 1.679;3.849 | <0.001 |
| Years of practice | 0.998 | 0.983;1.013 | 0.774 |
| Specialty level (Higher levels) | 0.678 | 0.505;0.909 | 0.009 |
| Type of hospital (private no teaching) | 0.358 | 0.146;0.878 | 0.025 |
| Hospital capacity (>400 beds) | 3.007 | 1.828;4.946 | <0.001 |
| Specialty surgery unit | 2.389 | 1.833;3.113 | <0.001 |
| Private practice | 0.777 | 0.598;1.009 | 0.059 |
| Involvement in research | 1.458 | 0.925;2.300 | 0.104 |
| Involvement in teaching | 1.282 | 0.892;1.842 | 0.180 |
| Number of articles published/year | 1.000 | 0.985;1.015 | 0.972 |
| Number of articles read/month | 0.993 | 0.983;1.002 | 0.146 |
| Number of conferences attended as speaker/year | 0.995 | 0.968;1.023 | 0.714 |
| Number of conferences attended as learner/year | 1.001 | 0.980:1.022 | 0.957 |
| Elective practice (beyond on-call) | 1.722 | 0.729;4.070 | 0.216 |
| On-calls in presence (76-100% of the cases) | 5.355 | 3.705;7.741 | <0.001 |
| Duration of the on-call shift | 1.365 | 0.940;1.983 | 0.101 |
| Number of weekly public holidays on call/month | 0.848 | 0.739;0.973 | 0.019 |

* Human Development Index
