# Supplementary material for: Global disparities in surgeons’ workloads, academic engagement and rest periods: the on-calL shIft fOr geNEral SurgeonS (LIONESS) study
Source: Updates Surg. 2024 Apr 29;76(5):1615–33. doi: 10.1007/s13304-024-01859-7 (PMC11455666; doi:10.1007/s13304-024-01859-7)
Supplement: Supplementary file 4 — Supplementary file4 Table 3. Results of the multivariable analysis of predictive factors of day-off after on-call (General population of responders). (DOC 15 KB) [file 13304_2024_1859_MOESM4_ESM.doc]

**Supplementary Material Table 3.** Results of the multivariable analysis of predictive factors of day-off after on-call.

| **Variable** | **Adjusted Odds Ratio (aOR)** | **95% Confidence Interval** | **P value** |
| --- | --- | --- | --- |
| HDI* (Very high and high) | 1.324 | 0.588;2.984 | 0.498 |
| HDI* (Very high) | 1.993 | 1.116;3.558 | 0.020 |
| Specialty level (Higher levels) | 0.713 | 0.524;0.971 | 0.032 |
| Type of hospital (private no teaching) | 0.752 | 0.435;1.301 | 0.309 |
| Hospital capacity (>400 beds) | 2.423 | 1.434;4.094 | <0.001 |
| Specialty surgery unit | 2.087 | 1.585;2.749 | <0.001 |
| On-call in presence | 5.446 | 3.651;8.125 | <0.001 |
| Number of on-call/month | 0.917 | 0.996;0.950 | <0.001 |
| Number of weekly public holidays on call/month | 1.014 | 0.869;1.183 | 0.858 |

* Human Development Index
