# Supplementary material for: Global disparities in surgeons’ workloads, academic engagement and rest periods: the on-calL shIft fOr geNEral SurgeonS (LIONESS) study
Source: Updates Surg. 2024 Apr 29;76(5):1615–33. doi: 10.1007/s13304-024-01859-7 (PMC11455666; doi:10.1007/s13304-024-01859-7)
Supplement: Supplementary file 5 — Supplementary file5 Table 4. Results of the multivariable analysis of predictive factors of day-off after on-call (Population of responders who perform extended on-calls > 12 h). (DOC 14 KB) [file 13304_2024_1859_MOESM5_ESM.doc]

**Supplementary Material Table 4.** Results of the multivariable analysis of predictive factors of day-off after an on-call >12 hours.

| **Variable** | **Adjusted Odds Ratio (aOR)** | **95% Confidence Interval** | **P value** |
| --- | --- | --- | --- |
| High HDI* | 0.846 | 0.268;2.670 | 0.776 |
| Very high HDI* | 2.148 | 1.101;4.190 | 0.025 |
| Specialty level (Trainee vs. Consultant/Professor) | 0.860 | 0.563;1.312 | 0.484 |
| N. on-calls/month | 0.857 | 0.810;0.906 | < 0.001 |
| In-house on-calls >50% | 4.297 | 2.352;7.852 | < 0.001 |
| In-house on-calls >75% | 1.669 | 0.969;2.874 | 0.065 |

* Human Development Index
